# Vps21 Directs the PI3K-PI(3)P-Atg21-Atg16 Module to Phagophores via Vps8 for Autophagy

**DOI:** 10.3390/ijms23179550

**Published:** 2022-08-23

**Authors:** Lei Zhao, Weiming You, Dan Sun, Hui Xu, Xia You, Haiqian Xu, Zulin Wu, Zhiping Xie, Yongheng Liang

**Affiliations:** 1 Key Laboratory of Agricultural Environmental Microbiology of Ministry of Agriculture, College of Life Sciences, Nanjing Agricultural University, Nanjing 210095, China; 2 School of Life Sciences and Technology, Shanghai Jiao Tong University, Shanghai 200240, China

**Keywords:** phosphatidylinositol 3-phosphate, the PI3K complex, endosomes, phagophore assembly site, Vps21, Vps8, Atg21

## Abstract

Phosphatidylinositol 3-phosphate (PI(3)P) serves important functions in endocytosis, phagocytosis, and autophagy. PI(3)P is generated by Vps34 of the class III phosphatidylinositol 3-kinase (PI3K) complex. The Vps34-PI3K complex can be divided into Vps34-PI3K class II (containing Vps38, endosomal) and Vps34-PI3K class I (containing Atg14, autophagosomal). Most PI(3)Ps are associated with endosomal membranes. In yeast, the endosomal localization of Vps34 and PI(3)P is tightly regulated by Vps21-module proteins. At yeast phagophore assembly site (PAS) or mammalian omegasomes, PI(3)P binds to WD-repeat protein interacting with phosphoinositide (WIPI) proteins to further recruit two conjugation systems, Atg5-Atg12·Atg16 and Atg8-PE (LC3-II), to initiate autophagy. However, the spatiotemporal regulation of PI(3)P during autophagy remains obscure. Therefore, in this study, we determined the effect of Vps21 on localization and interactions of Vps8, Vps34, Atg21, Atg8, and Atg16 upon autophagy induction. The results showed that Vps21 was required for successive colocalizations and interactions of Vps8-Vps34 and Vps34-Atg21 on endosomes, and Atg21-Atg8/Atg16 on the PAS. In addition to disrupted localization of the PI3K complex II subunits Vps34 and Vps38 on endosomes, the localization of the PI3K complex I subunits Vps34 and Atg14, as well as Atg21, was partly disrupted from the PAS in *vps21*∆ cells. The impaired PI3K-PI(3)P-Atg21-Atg16 axis in *vps21*∆ cells might delay autophagy, which is consistent with the delay of early autophagy when Atg21 was absent. This study provides the first insight into the upstream sequential regulation of the PI3K-PI(3)P-Atg21-Atg16 module by Vps21 in autophagy.

## 1. Introduction

Autophagy is a progressive process in which unwanted materials are enclosed in autophagosomes (APs), followed by the fusion of outer membranes of APs and lysosomes/vacuoles, which delivers the unwanted materials into lysosomes/vacuoles for subsequent degradation [1,2,3]. This progressive process requires the class III phosphatidylinositol 3-kinase (PI3K) complex I and phosphatidylinositol 3-phosphate (PI(3)P) during the autophagy initial stage, while the endosomal sorting complex required for transport (ESCRT) is necessary during the later autophagosome closure stage [4]. Phosphoinositides (PIs) function in almost all cellular physiological processes, especially in intracellular membrane trafficking events, including autophagy. Kinases and phosphatases alter the phosphorylation states of PIs in the inositol headgroup to generate distinct PIs [5,6,7]. PI(3)P is normally generated from PI molecules by the Vps34 subunit of PI3K complex [8,9]. However, Vps34 is a common subunit of the two forms of PI3K complexes that are distinguished by Vps38/UVRAG as a PI3K complex II-specific subunit or Atg14/ATG14L as a PI3K complex I-specific subunit [10]. PI(3)P generated by PI3K complex II on endosomes [11] plays a significant role in endocytosis, whereas PI(3)P generated by PI3K complex I at yeast phagophore assembly sites (PAS) or mammalian omegasomes is important for autophagy [10,12]. At the PAS or omegasomes, PI(3)P binds to WD-repeat protein interacting with phosphoinositide (WIPI) proteins to further recruit two conjugation systems, Atg12-Atg5·Atg16 and Atg8-PE (LC3-II), which initiate and fulfill the autophagy process [13,14]. Under normal growth conditions, the localizations of PI(3)P and Vps34 on endosomes are tightly regulated by Rab5/Vps21 in mammalian and yeast cells [15,16]. However, whether Rab5/Vps21 regulates PI3K complex and PI(3)P localization to the PAS or omegasomes, and how the absence of such localization affects autophagy remain obscure.

Yeast cells express three PI(3)P-binding proteins, while mammalian cells express at least four WIPI proteins [17]. The yeast Atg21 and mammalian WIPI2 proteins are frequently studied in autophagy as PI(3)P-binding proteins. The binding of Atg21 at the PAS is dependent on PI(3)P [18]. Furthermore, Atg21 interacts with a few Atg proteins, including Atg16 and Atg8, in order to carry out autophagy [18]. As Vps34 and PI(3)P have previously been found to be mislocalized in a Rab5/Vps21 mutant cell line [15,16], it is possible that the localizations and functions of their effectors are also affected.

Vps21-module proteins comprise the guanine nucleotide-exchange factor (GEF) Vps9, the Rab GTPase Vps21, the tether class C core vacuole/endosome tethering (CORVET) complex with specific subunits Vps3 and Vps8, and other effectors (Vac1, Pep12, and Vps45). Ypt7-module proteins comprise corresponding proteins, including the homotypic fusion and vacuole protein sorting (HOPS) complex with specific subunits Vps39 and Vps41. These module proteins function in both vesicle trafficking and autophagy [19,20]. In this study, we screened for interactions between Vps21-module proteins and PI3K complex subunits and found that Vps8 (but not Vps3) interacted with Vps34, which further interacted with Atg21 under the regulation of Vps21. Furthermore, the interaction between Atg21 and Atg8 or Atg16 was also found to be Vps21-dependent. The absence of Atg21 or Vps21 led to a significant decrease in the localization of Atg5 and Atg16 on the PAS. We propose that Vps21 regulates autophagy partly through the PI3K-PI(3)P-Atg21-Atg16 axis in progressing autophagy in the early stage and through the ESCRT complex in closing phagophores (unclosed APs) in the later stage.

## 2. Results

### 2.1. The Vps21 Module Regulates Vps34 and PI(3)P Localizations under Nitrogen Starvation

Previously, we reported that yeast ESCRT complex regulates phagophore closure in a Vps21-dependent manner under short-term nitrogen starvation (SD-N) conditions [20,21,22]. However, under normal growth conditions, the absence of Vps21 and its GEF Vps9 resulted in mislocalized Vps34 and PI(3)P [16]. In this study, we investigated whether the mislocalized Vps34 and PI(3)P in Vps21-module mutants contribute to autophagic defects under nitrogen starvation and, if so, how this is accomplished mechanistically.

When PI(3)P was probed with DsRed-FYVE, FYVE bound to endosomes and vacuoles in wild-type (WT) cells, although the signal on vacuoles may not be as strong as when it is punctate on endosomes [23,24]. We probed PI(3)P with DsRed-FYVE and confirmed that Vps34 and PI(3)P were mislocalized in Vps21-module mutant cells under normal growth conditions. The localizations of Vps34 and PI(3)P changed from a dominant punctate pattern in cytosol in wild-type (WT) cells to a dominant ring pattern with dots on vacuolar membranes in Vps21-module mutant cells (Figure S1A). These observations were in agreement with previous findings [16]. We further found that Vps34 and PI(3)P mislocalized in Vps21-module mutant cells but not in Ypt7 mutant cells in SD-N medium. The percentage of cells with a FYVE ring on vacuoles increased from approximately 10% in WT and *ypt7*∆ cells to 30–60% in Vps21-module mutant cells. PI(3)P was not detected in *vps15*∆ cells and showed a diffused pattern in *vps34*∆ cells (Figure S1A,B). To determine whether Vps21-module proteins affect the localizations of both PI3K complexes, we examined the localizations of PI3K complex-specific subunits in representative Vps21-module mutants. The results showed that Vps38 puncta decreased in Vps21-module mutant cells, whereas Atg14 localization did not change in the same Vps21-module mutant (Figure S1C). However, Vps21 depletion did not affect the protein levels of Vps38, Vps34, and Atg14 in cells cultured in SD-N medium (Figure S1D). In addition, Vps34 and PI(3)P mislocalization in representative Vps21-module mutants, *vps21*∆ and *vps8*∆, was suppressed by overexpressing Vps21 (the WT and GTP-bound form but not the GDP-bound form) and Vps8, respectively (Figure S1E,F). In contrast, Vps21 localization to mCherry-Atg8-labeled PAS was enhanced in the *vps34*∆ mutant, from approximately 30% in WT cells to 70% in *vps34*∆ mutant cells. (Figure S1G). These results suggest that the Vps21 module recruited PI3K complex II to endosomes to generate PI(3)P under nitrogen starvation.

### 2.2. Vps8 Interacts with Vps34 on Endosomes and the PAS under Nitrogen Starvation, and This Interaction Depends on Vps21

**Figure 1:**
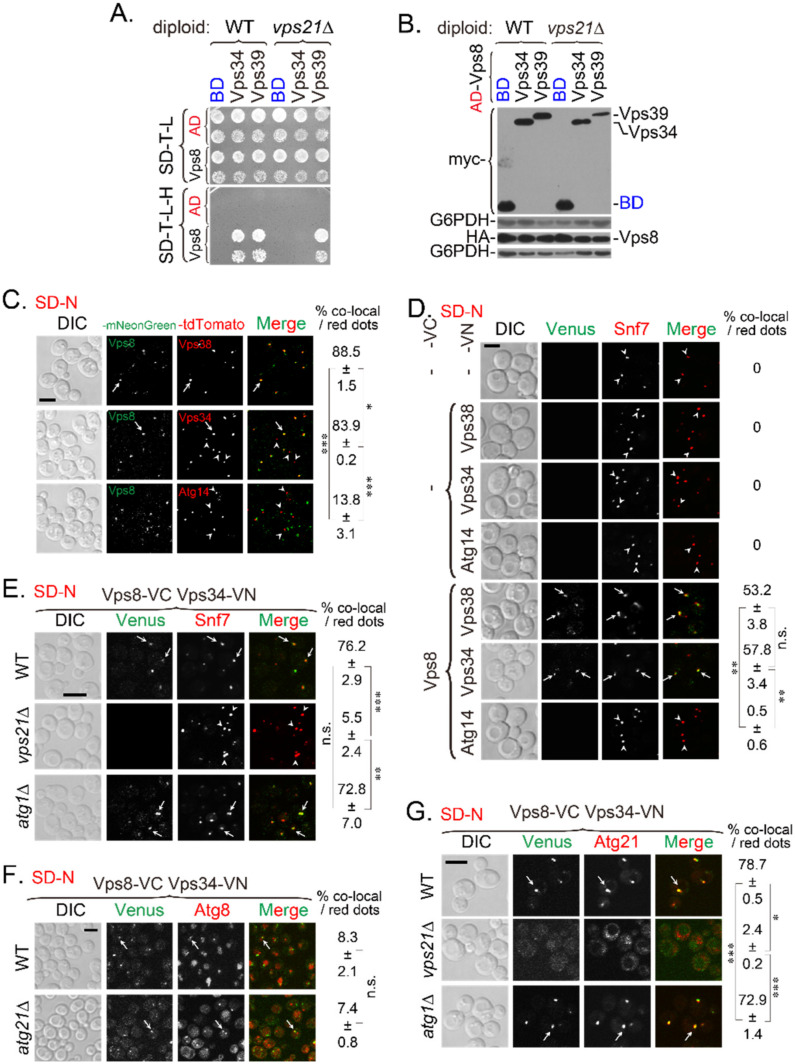
Vps8 interacts with Vps34 on endosomes under nitrogen starvation in a Vps21-dependent manner. (**A**) Y2H assay showed that the Vps8-Vps34 interaction depended on Vps21. Vps8 was expressed from the pACT2 (AD) vector, and Vps34 and Vps39 were expressed from the pGBKT7 (BD) vector. Vps8 interacted with Vps34 in wild-type (WT) cells, but not in *vps21*∆ cells. The Vps21-independent interaction between Vps8 and Vps39 served as a control. (**B**) Immunoblotting analysis indicated that Vps8 and Vps34 were expressed in *vps21*∆ cells. Diploid cells (panel **A**) grown on SD-Trp-Leu (SD-T-L) plates were analyzed for protein expression with an anti-HA antibody (for those harboring pGADT7/pACT2 plasmids) or an anti-myc antibody (for those harboring pGBKT7 plasmids). G6PDH served as a loading control. (**C**) Vps8 colocalized with Vps38 and Vps34 under nitrogen starvation. The C-terminus of the Vps8 was tagged with mNeonGreen, and the C-termini of representative PI3K complex subunits (Vps38, Vps34, and Atg14) were tagged with tdTomato. Cells were grown in rich (YPD) medium to mid-log phase before being switched to nitrogen-starvation medium (SD-N) for 2 h to determine colocalization. (**D**) Vps8 interacted with Vps38 and Vps34 on Snf7-labeled endosomes under nitrogen starvation. The strains expressing Snf7-mCherry were further engineered to express proteins with BiFC tags (i.e., a C-terminal Venus [VC] tag or an N-terminal Venus [VN] tag), as indicated. These cells were analyzed for fluorescence, as described for panel (**C**). (**E**,**F**) The Vps8-Vps34 interaction depended on Vps21, as determined by BiFC assays. The strain expressing Vps8-VC and Vps34-VN was further tagged with Snf7-mCherry (**E**) or mCherry-Atg8 (**F**). *VPS21*, *ATG1*, or *ATG21* were deleted from the tagged strains. The indicated strains were grown and analyzed for fluorescence, as described for panel (**C**). (**E**) The Vps8-Vps34 interaction on Snf7-mCherry-labeled endosomes depended on Vps21. Vps8 interacted strongly with Vps34 on Snf7-mCherry-labeled endosomes in WT and *atg1*∆ cells but weakly in *vps21*∆ cells. (**F**) Weak Vps8-Vps34 interactions were observed at mCherry-Atg8-labeled phagophore-assembly site (PAS) and autophagosomes (APs) in WT and *atg21*∆ cells. (**G**) The Vps8-Vps34 interaction on Atg21-positive structures depended on Vps21, as did the puncta localization of Atg21. Vps8 interacted with Vps34 on Atg21-tdTomato-labeled structures (endosomes) in WT and *atg1*∆ cells. The Vps8-Vps34 interaction and the puncta localization of Atg21 disappeared in *vps21*∆ cells. In panels (**C**–**G**), the arrows indicate colocalizing puncta, and the arrowheads indicate puncta without colocalizing green puncta. Scale bars were 5 μm. The percentage of co-localizing dots (based on red dots) is expressed as the mean ± SD. Statistical significance was analyzed using Student’s *t*-test (n ≥ 2 experiments). n.s., not significant; *, *p* < 0.05; **, *p* < 0.01; ***, *p* < 0.001.

To explore the molecular mechanism wherein Vps34 and PI(3)P disappeared in Vps21-module mutants, we first screened for interactions between Vps21-module proteins and PI3K complex subunits by performing a yeast two hybrid (Y2H) assay. We found that Vps8 interacted with Vps34 (Figure 1A) and confirmed that these Y2H plasmids were functional (Figure S7A–C). However, this interaction disappeared in the absence of Vps21 (Figure 1A). The protein expression results from Y2H strains indicated that the lost interaction between Vps8 and Vps34 in *vps21*∆ cells was not due to decreased or no Vps8 expression, or decreased Vps34 expression, as Vps8 and Vps39 still interacted in *vps21*∆ cells when Vps39 expression was decreased further (Figure 1A,B). Vps39 is a homotypic fusion and vacuole protein sorting (HOPS) complex-specific subunit [25]. The physiological relevance of our new finding of Vps21-independent Vps8-Vps39 interaction should be further investigated in the future.

Second, we determined the colocalization between Vps8 and representative PI3K subunits by tagging PI3K subunits at the C-terminus with tdTomato and tagging Vps8 at the C-terminus with mNeonGreen through chromosomal modifications. Colocalization analysis of cells cultured in SD-N medium showed that Vps8 colocalized with Vps38 and Vps34 in over 80% of the tdTomato dots but with Atg14 in only about 10% of tdTomato dots (Figure 1C). These results indicate that Vps8 might associate mainly with the PI3K complex II on endosomes.

Third, we determined the interaction between Vps8 and representative PI3K subunits by performing bimolecular fluorescence complementation (BiFC) assays with endogenously expressed proteins. Considering that the subunits of the PI3K complex II mainly localize to endosomes, and Snf7 mainly localizes to endosomes although a small portion of Snf7 was recruited to the PAS during autophagy, we could still use Snf7-mCherry as an endosomal marker [22,26] to determine whether Vps8 interacted with PI3K complexes on endosomes. Vps8 interacted with Vps38 and Vps34 in over 50% of Snf7-mCherry dots but with Atg14 in only about 1% of Snf7-mCherry dots (Figure 1D). We further found that the Vps8-Vps34 interaction occurred in Snf7-mCherry-labeled endosomes in WT and *atg1*∆ cells in over 70% of Snf7-mCherry dots but disappeared in *vps21*∆ cells in only about 5% of Snf7-mCherry dots (Figure 1E), indicating that this interaction was Vps21-dependent but autophagy-independent. As Vps34 localized at both endosomes and the PAS, we questioned whether the Vps8-Vps34 interaction also partly occurred on the PAS. We used mCherry-Atg8 as a PAS marker, although it might also be an AP marker in WT cells. The results showed that Vps8 interacted with Vps34 in about 7–8% of mCherry-Atg8 dots in both WT and *atg21*∆ cells. The *atg21*∆ cells were intentionally used to increase the percentage of cells with mCherry-Atg8 dots (Figure 1F).

Atg21 binds to PI(3)P, which is a product of Vps34 [8]. Atg21 is largely localized to endosomes; thus, it can serve as an endosomal marker in WT cells [26]. When Atg21-tdTomato was used as an endosomal marker, the Vps8-Vps34 interaction also occurred on Atg21-labeled dots in WT and *atg1*∆ cells grown in SD-N medium in over 70% of Atg21-tdTomato dots, but not in *vps21*∆ cells in only about 2% of Atg21-tdTomato dots. Furthermore, Atg21 diffused in the cytosol of *vps21*∆ cells but not in that of *atg1*∆ cells (Figure 1G). The endosomal marker, Snf7-mCherry, did not disappear in *vps21*∆ cells as Atg21-tdTomato did, indicating that the lost interaction between Vps8 and Vps34 in *vps21*∆ cells was not due to endosome dismantling. Furthermore, Vps8 interacted with Vps34 in WT cells but not in *vps21*∆ cells, regardless of which red fluorescence protein was detected in background cells (Figure 1E–G). We deduced that no interaction occurred between Vps8 and Vps34 in *vps21*∆ cells, and almost no colocalization occurred in mCherry-Atg8 dots. Together, these results indicate that Vps21 controlled the Vps8-Vps34 interaction on endosomes and the PAS under nitrogen starvation.

### 2.3. The Vps34-Atg21 Interaction on Endosomes Depends on Vps21

**Figure 2:**
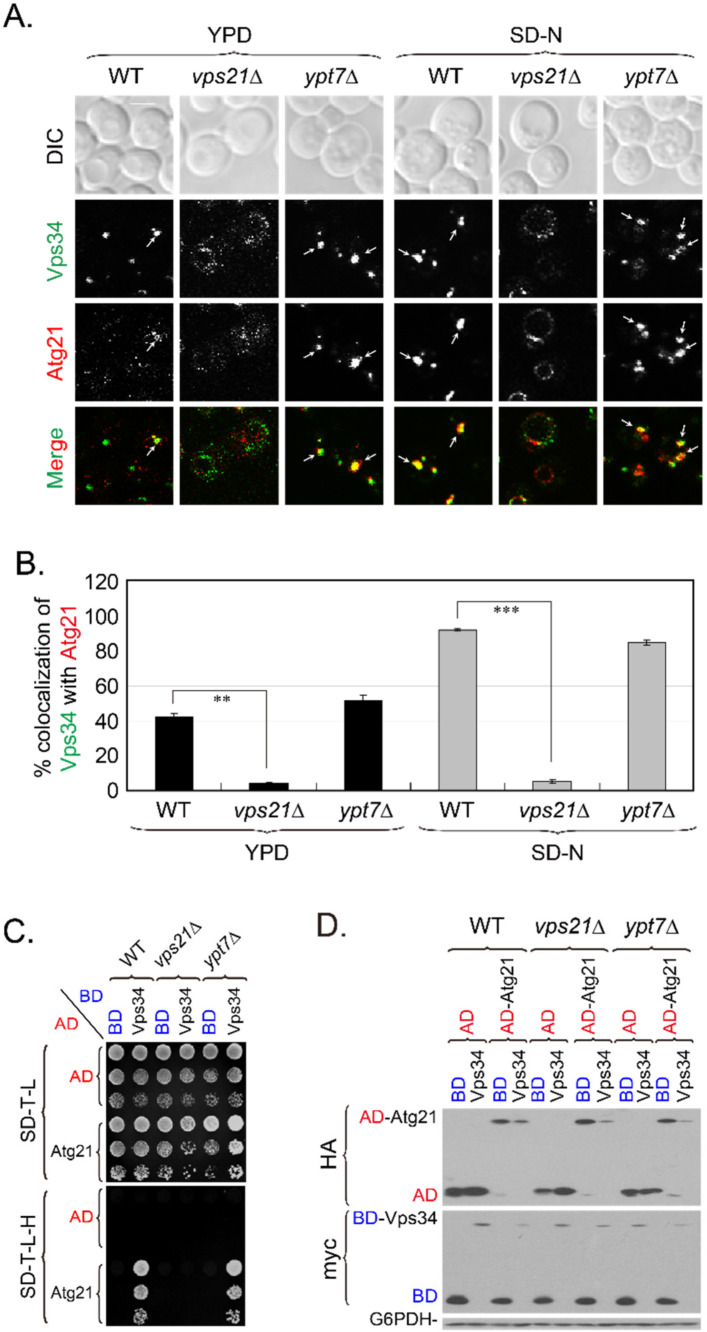
Vps34 interacts with Atg21 in a Vps21-dependent manner. (**A**) The colocalization between Vps34 and Atg21 depended on Vps21 but not on Ypt7. *VPS21* or *YPT7* was deleted from the Vps34-mNeonGreen and Atg21-tdTomato tagged strains. The indicated strains were grown in YPD medium, or further grown in SD-N medium for 2 h for fluorescence determinations. The arrows indicate colocalizing puncta; scale bar, 2 μm. (**B**) Quantification of the Vps34-Atg21 colocalization shown in panel (**A**). The percentage of colocalizing dots (based on Atg21-positive dots) was quantified from at least 70 red dots in two independent experiments and is expressed as the mean ± SD. **, *p* < 0.01; ***, *p* < 0.001. (**C**) The Vps34-Atg21 interaction depended on Vps21 but not on Ypt7, as determined by Y2H assays. The interactions were determined in WT, *vps21*∆, and *ypt7*∆ cells. (**D**) Immunoblotting analysis indicated that Vps34 and Atg21 were expressed at similar levels in *vps21*∆ cells as in WT and *ypt7*∆ cells. The diploid cells shown in panel (**C**) (grown on SD-T-L plates) were analyzed for protein expression as in Figure 1B. G6PDH served as a loading control.

The loss of interaction between Vps8 and Vps34 on endosomes and the PAS in *vps21*∆ cells grown in SD-N medium could be attributed to mislocalizations of Vps8 (Figure S2A,B and [27]) and Vps34 (Figures S1 and S2C,D and [16]). Vps34 mislocalization in *vps21*∆ cells might further affect the localization and/or interaction of its effectors/interactors, such as Atg21. To further characterize the relationship between Vps34 and Atg21 in *vps21*∆ cells (Figure 1G), we examined the colocalization between Vps34-mNeonGreen and Atg21-tdTomato. Vps34 colocalized with Atg21 in WT and *ypt7*∆ cells in rich (that is, yeast extract–peptone–dextrose, YPD) medium in 40–50% of Atg21-tdTomato dots, and these colocalizations were enhanced in SD-N medium in over 90% of Atg21-tdTomato dots. However, the colocalizations were abolished in *vps21*∆ cells in only about 5% of Atg21-tdTomato dots (Figure 2A,B). To determine whether the colocalization of Vps34 and Atg21 reflected interactions, we assayed for potential interactions between them by performing a Y2H assay, after which we confirmed that the Y2H plasmids were functional (Figure S7B,D). Vps34 strongly interacted with Atg21 in WT and *ypt7*∆ cells, but this interaction was completely abolished in *vps21*∆ cells (Figure 2C). However, this abolishment was not due to decreased protein expression levels of Vps34 and Atg21 in *vps21*∆ cells, as their levels were equal to those in WT and *ypt7*∆ cells (Figure 2D). The normal interaction and colocalization between Vps34 and Atg21 in *ypt7*∆ cells indicated that the defective interaction and colocalization between Vps34 and Atg21 in *vps21*∆ cells was not due to a general defect in endocytosis.

To dissect the hierarchy among Vps21, Vps8, Vps34, and Atg21 on endosomes, we analyzed the colocalizations of these proteins on Snf7-mCherry-labeled endosomes in different strains grown in YPD or SD-N medium. Vps21 dots decreased or showed a diffused pattern in *vps8*∆ cells grown in YPD medium [19]. Regardless of whether cells were grown in YPD or SD-N medium, Vps8 localized to endosomes in WT and *ypt7*∆ cells in around 80% of Snf7-mCherry dots but diffused into the cytosol in *vps21*∆ cells and localized to endosomes in less than 10% of Snf7-mCherry dots (Figure S2A,B). The Vps8 diffusion is consistent with the soluble fractionation of Vps8 observed previously in *vps21*∆ cells when the protein level of Vps8 did not decrease prevalently in *vps21*∆ cells [28]. Vps8 did not diffuse but localization on endosomes in *vps34*∆ cells was only in about 20% of Snf7-mCherry dots (Figure S2A,B), whereas Vps34 changed from a dominant endosomal puncta pattern in WT cells to a dominant ring with occasional dots on vacuolar membranes pattern in *vps8*∆ cells (Figure S1A,B). These results indicate that Vps8 and Vps34 affected each other’s localization. Consistently, Vps34 localized to endosomes in *vps21*∆ cells grown in YPD or SD-N medium only in about 10% of Snf7-mCherry dots (Figure 2A, Figures S1A and S2C,D and [16]), whereas Vps34 localized on endosomes in WT and *ypt7*∆ cells in around 80% of Snf7-mCherry dots (Figures S1 and S2C,D).

We also found that Vps34 localized to endosomes in *atg21*∆ cells grown in YPD or SD-N medium in around 80% of Snf7-mCherry dots (Figure S2C,D). In contrast, Atg21 was diffused in cytosol and localized to endosomes in *vps34*∆ and *vps21*∆ cells only in about 20% of Snf7-mCherry dots (Figure S2E,F), which also occurred in *vps8*∆ cells [26]. These results, together with the observation that Vps21 did not disappear in *vps34*∆ cells (Figure S1G), indicate that Vps21 dominated the localizations of Vps8, Vps34, and Atg21; and Vps8 dominated the localization of Vps34 and Atg21; and that Vps34 dominated the localization of Atg21 (not the opposite). We propose that the Vps21-dependent Vps8-Vps34 interaction on endosomes is important for the recruitment of Vps34, which in turn recruits Atg21 to endosomes. In the absence of Vps21, both Vps34 and Atg21 disappeared from endosomes. The localizations of Vps8 and Vps34 on endosomes or their interaction are important for Atg21 localization to endosomes.

### 2.4. PI3K Complex II Subunits Mainly Localize to Endosomes and Partially Localize to the PAS, Depending on Vps21

Vps34 is a common subunit of PI3K complexes I and II, and these complexes participate in vesicle trafficking and autophagy, respectively [10]. Vps34 is thought to be localized to both endosomes and the PAS. The absence of Vps21 affects the localization of Vps34 (Figure 2A, Figures S1A and S2C). Thus, it is important to determine the Vps34-localization changes at endosomes and the PAS in the same *vps21*∆ cells. It is also important to determine changes in the localization of the PI3K complex-specific subunits Vps38 and Atg14 to endosomes and the PAS in *vps21*∆ cells.

We first examined colocalization changes in the PI3K complex II-specific subunit Vps38 at Snf7-mCherry-labeled endosomes in related strains. We found that Vps38-Snf7 colocalization was greatly enhanced with nitrogen starvation unless *VPS21* was deleted. The depletion of Atg proteins did not change the colocalization of Vps38 and Snf7. In the presence of Vps21, the percentage of cells showing Vps38-Snf7 colocalization increased from approximately 60% in YPD medium to more than 90% in SD-N medium, if Vps21 was present. However, this percentage was less than 10% in the absence of Vps21 (Figure S3). Similarly to Vps38 localization, Vps34-Snf7 colocalization depended on Vps21 and nitrogen starvation but not on autophagy, as depleting representative Atg proteins did not alter the colocalization (Figure S4). As expected, most of the PI3K complex I-specific subunit Atg14 did not colocalize with Snf7 in related strains. However, the colocalization of Atg14 and Snf7 increased under nitrogen starvation in 3–5% of Snf7-mCherry dots and remarkably decreased in less than 3% of Snf7-mCherry dots if *VPS21* was deleted, whereas co-depleting Atg8 and Atg21 slightly decreased this colocalization (Figure S5).

We examined the colocalizations of representative PI3K complex subunits with RFP-Ape1, which labeled the PAS (and potentially APs in WT cells) under nitrogen starvation. As expected, a higher percentage of cells showed Ape1 colocalization with Vps34 or Atg14 than with Vps38. Vps34-Ape1 and Atg14-Ape1 colocalization was in more than 30% of PAS dots while Vps38-Ape1 colocalization was only in less than 20% of PAS dots in WT cells. The localization of Vps38 on the PAS was low but definite. All colocalizations were significantly lower in the absence of Vps21 in less than 10% of PAS dots in *vps21*∆ cells. The percentage of cells showing colocalization of Vps34 or Atg14 with Ape1 was higher when Atg8 was depleted from WT cells, resulting in more visible PAS dots. Vps34-Ape1 and Atg14-Ape1 colocalization peaked in 70% of PAS dots while Vps38-Ape1 colocalization peaked only in 20% of PAS dots in *atg8*∆ cells. (Figure S6).

**Figure 3:**
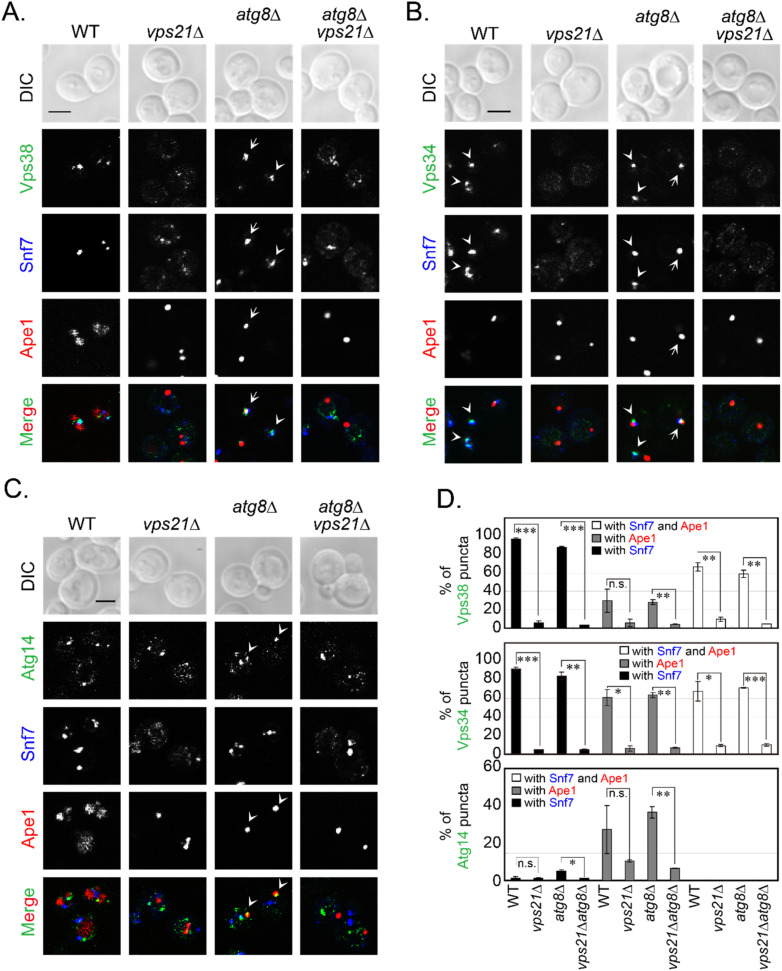
Localizations of PI3K complex subunits on endosomes and/or the PAS decrease in *vps21*∆ cells under nitrogen starvation. Cells were grown to mid-log phase and then switched to SD-N medium for 2 h. (**A**) Vps38-mNeonGreen, mainly localized to Snf7-2xmTagBFP2-labeled endosomes, although it also partially localized to RFP-Ape1-labeled PAS/APs and to dots positive for both Snf7-2xmTagBFP2 and RFP-Ape1. (**B**) Vps34-mNeonGreen largely localized to Snf7-2xmTagBFP2-labeled endosomes, although it also considerably localized to RFP-Ape1-labeled PAS/APs and to over half of all dots that were positive for both Snf7-2xmTagBFP2 and RFP-Ape1. (**C**) Atg14-2xmNeonGreen, partially localized to RFP-Ape1-labeled PAS/APs, and it also weakly localized to Snf7-2xmTagBFP2-labeled endosomes but did not localize to dots positive for both Snf7-2xmTagBFP2 and RFP-Ape1. In panels (**A**–**C**), all colocalizations were Vps21-dependent. The arrows indicate colocalization of the PI3K complex subunit at dots positive for both Snf7-2xmTagBFP2 and RFP-Ape1. The arrowheads indicate colocalization of the PI3K complex subunit at either endosomes or the PAS/APs. Scale bars, 2 μm. (**D**) Quantification of the subunits of PI3K complexes at endosomes and/or the PAS/APs. The percentages of green puncta (Vps38, top; Vps34, middle; Atg14, bottom) at Snf7-2xmTagBFP2-labeled endosomes, RFP-Ape1-labeled PAS/APs, or both (using samples represented in panels (**A**–**C**)) were quantified and are presented as the mean ± SD. n.s., not significant; *, *p* < 0.05; **, *p* < 0.01; ***, *p* < 0.001.

Since representative PI3K complex subunits (Vps34, Vps38, and Atg14) colocalized with Snf7-mCherry and RFP-Ape1 to some degree, we expected that some PI3K complex subunits would simultaneously colocalize with Snf7-mCherry and RFP-Ape1. To test this possibility, we labeled endosomes and the PAS in the same cells and then checked for colocalizations of the PI3K complex subunit proteins with these markers. Endosomes were labeled with Snf7-2xmTagBFP2, which was functional as it complemented the defects of GFP-Atg8 fluorescence and degradation in *snf7*∆ cells (Figure S7E,F). We compared these colocalizations in WT and *vps21*∆ cells with or without Atg8. Labeled PAS dots were more prevalent when Atg8 was absent. Vps38 localization to Snf7-labeled endosomes was over 80% in WT and *atg8*∆ cells, whereas Vps38 localization to Ape1-labeled PAS was only approximately 30%. However, approximately 60% of Snf7 and Ape1 colocalization dots were Vps38-positive in WT and *atg8*∆ cells. In contrast, all such colocalizations significantly decreased to less than 10% when Vps21 was absent, regardless of whether Atg8 was present (Figure 3A,D).

Similarly, Vps34 localization to Snf7-labeled endosomes was over 80% in WT and *atg8*∆ cells, and Vps34 localization to Ape1-labeled PAS was over 60%. More than 60% of Snf7 and Ape1 colocalization dots were Vps34-positive in WT and *atg8*∆ cells. In contrast, all such colocalizations significantly decreased to less than 10% in the absence of Vps21, regardless of whether Atg8 was present (Figure 3B,D). Atg14 localization to Snf7-labeled endosomes was less than 10% in WT and *atg8*∆ cells, although Atg14 localization to Ape1-labeled PAS increased to approximately 30%. However, almost no Snf7 and Ape1 colocalization dots were Atg14-positive in WT and *atg8*∆ cells. In contrast, the colocalization of Atg14 to Snf7-labeled endosomes or Ape1-labeled PAS significantly decreased when both Vps21 and Atg8 were absent (Figure 3C,D). These results are consistent with separate measurements of PI3K complex subunit proteins on endosomes or the PAS in the same background strains (Figure S3–S6).

### 2.5. Atg21 Mainly Localizes to Endosomes and Also to the PAS, in a Vps21-Dependent Manner

**Figure 4:**
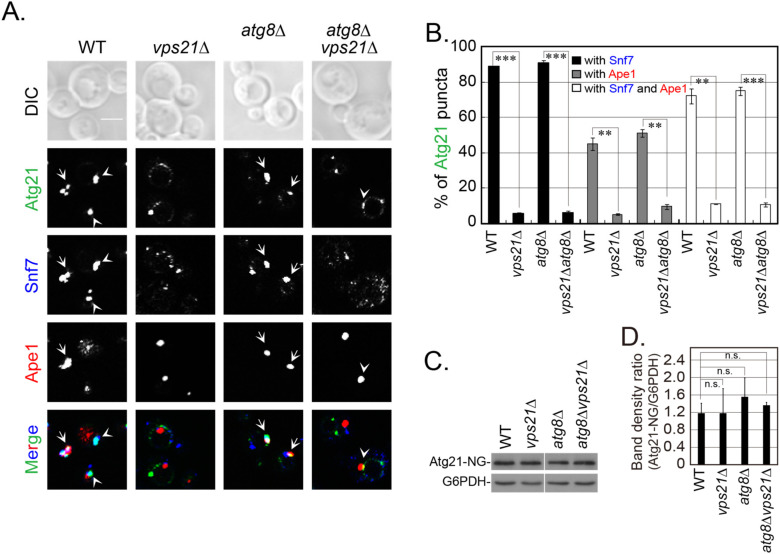
Atg21 localization to endosomes and/or the PAS decreases in *vps21*∆ cells, and both Atg21 and Snf7 are dispersed under nitrogen starvation. Cells were grown and treated as described in Figure 3. (**A**) Atg21-mNeonGreen mainly localized at Snf7-2xmTagBFP2-labeled endosomes, although it also showed partial localization to RFP-Ape1-labeled PAS/APs and also localized heavily to dots positive for both Snf7-2xmTagBFP2 and RFP-Ape1. All colocalizations were Vps21-dependent. The arrows indicate simultaneously colocalization of Atg21 at both endosomes and the PAS/APs, and the arrowheads indicate Atg21 localization to either endosomes or the PAS. Scale bar, 2 μm. (**B**) Quantification of Atg21 at endosomes and/or the PAS/APs. The percentages of Atg21-positive puncta at Snf7-2xmTagBFP2-labeled endosomes and/or RFP-Ape1-labeled PAS/APs (panel **A**) were quantified and are presented as the mean ± SD. (**C**) The protein expression levels of Atg21 in wild-type and *vps21*∆ cells were equal, regardless of whether *ATG8* was deleted. An anti-mNeonGreen antibody was used to probe for Atg21-mNeonGreen (NG). G6PDH served as a loading control. (**D**) Quantification of the Atg21-mNeonGreen: G6PDH band-density ratios (panel **C**). The data are presented as the mean ± SD. Panels (**B**,**D**): n.s., not significant, **, *p* < 0.01; ***, *p* < 0.001.

The absence of Vps21 resulted in the mislocalization of PI3K complex subunits. Furthermore, Atg21, which binds to PI(3)P (the product of Vps34) also mislocalized in *vps21*∆ cells (Figure 1G and Figure 2A,B). To closely study localization changes in Atg21-mNeonGreen from endosomes and the PAS, we labeled endosomes and/or the PAS with fluorescent proteins and examined colocalizations for cells grown in SD-N medium for 2 h. When endosomes were labeled with Snf7-mCherry, Atg21 showed high colocalization with approximately 90% of Snf7 dots in WT cells and related mutants, except for *vps21*∆ cells. However, this colocalization frequency significantly decreased to less than 10% in *vps21*∆ and *vps21*∆*atg8*∆ cells (Figure S8A,C). The colocalization pattern of Atg21 with Snf7 was similar to those of Vps34 and Vps38 with Snf7 (Figures S3 and S4). Atg21 colocalized less to RFP-Ape1-labeled PAS than Snf7-mCherry-labeled endosomes in the same strain, with a maximum colocalization of approximately 70% found in *atg8*∆ cells. Similarly, Atg21 localization to the PAS was lower than 20% of PAS dots in the absence of Vps21 (Figure S8B,D). To investigate changes in the colocalization of Atg21 to endosomes and the PAS in the same cell, we labeled the same strain with Snf7-2xmTagBFP2 and RFP-Ape1. Consistently, Atg21 mainly colocalized with Snf7-2xmTagBFP2 in approximately 90% of Snf7 dots and with RFP-Ape1 in approximately 40–50% of Ape1 dots in both WT and *atg8*∆ cells. However, Atg21 colocalized with approximately 70% of dots positive for both Snf7-2xmTagBFP2 and RFP-Ape1. Nevertheless, Atg21 significantly mislocalized from dots with Snf7-2xmTagBFP2 and RFP-Ape1 colocalization to be less than 10% of these colocalization dots when Vps21 was depleted from WT or *atg8*∆ cells (Figure 4A,B). We also determined the protein expression level of Atg21-mNeonGreen in these strains. Our results showed that the Atg21 mislocalization was not due to changes in the Atg21 protein levels as the ratios are around 1-fold in all strains (Figure 4C,D). The change in the Atg21-localization pattern in the tested strains was similar to those of Vps34 and Vps38. Together, these findings indicate that Vps21 controlled the Vps34 and Atg21 recruitment to endosomes, which determined subsequent interactions between Vps34 and Atg21 on endosomes (Figure 2).

### 2.6. Interactions of Atg21 with Atg16 and Atg8 Depend on Vps21 and PI3K Complex II

**Figure 5:**
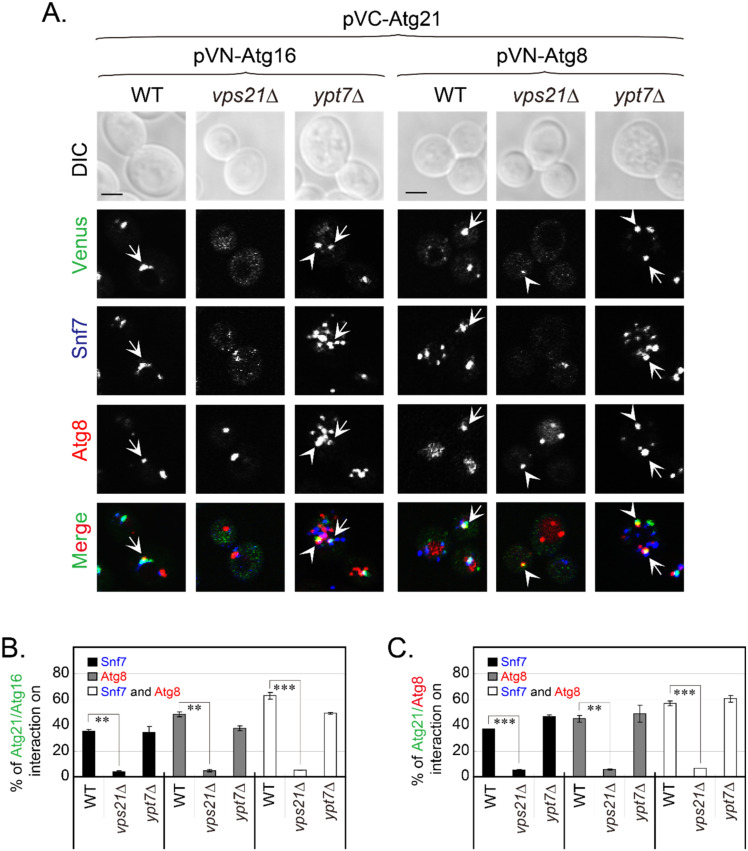
Atg21 interacts with Atg16 and Atg8 in a Vps21-dependent manner. (**A**) Atg21 interacted with Atg16 (left) and Atg8 (right) on Snf7-2xmTagBFP2-labeled endosomes and mCherry-Atg8-labeled PAS/APs under nitrogen starvation, and these interactions were Vps21-dependent. The indicated BiFC plasmids were transformed into WT, *vps21*∆, and *ypt7*∆ cells expressing Snf7-2xmTagBFP2 and mCherry-Atg8, grown, and analyzed for fluorescence, as described in Figure 3A, except that the cells were under nitrogen starvation for 30 min to visualize Atg8-positive dots. Atg21 interacted with Atg16 (left) and Atg8 (right) on endosomes and the PAS/APs in WT and *ypt7*∆ cells, but not in *vps21*∆ cells. The arrows indicate simultaneous interactions on endosomes and the PAS/APs, and the arrowheads indicate interactions on the PAS/APs. Scale bars, 2 μm. (**B**,**C**) Quantification of Atg21 interactions on endosomes and/or the PAS/APs with Atg16 (**B**) or Atg8 (**C**). The percentages of Venus-positive puncta (interactions) on endosomes and/or the PAS/APs (panel **A**) were quantified and are presented as the mean ± SD. **, *p* < 0.01; ***, *p* < 0.001.

Atg21 plays roles in autophagy partly through interacting with Atg16 and Atg8 [18]. Atg21 mislocalized from endosomes and the PAS in *vps21*∆ cells, which most likely affected the interactions of Atg21 with Atg16 and Atg8 at both sites. We used BiFC assays to confirm the interactions between Atg21 and Atg16 or Atg8. We verified the functions of the constructed BiFC plasmids with complementary assay (Figure S9A–C). By using Snf7-2xmTagBFP2 as an endosomal marker and mCherry-Atg8 as a PAS marker, we found that the Atg21-Atg16 interaction occurred on endosomes and/or the PAS. In WT cells, Atg21-Atg16 interaction occurred on approximately 30% of endosomes, 50% of the PAS, and 60% of Snf7 and Atg8 colocalization dots. However, these interactions and colocalizations significantly decreased to less than 5% of all in *vps21*∆ cells but did not decrease in *ypt7*∆ cells (Figure 5A,B). The interactions and colocalizations of Atg21 and Atg8 on endosomes and/or the PAS were quite similar to those of Atg21 and Atg16 in the same background strain (Figure 5A,C).

**Figure 6:**
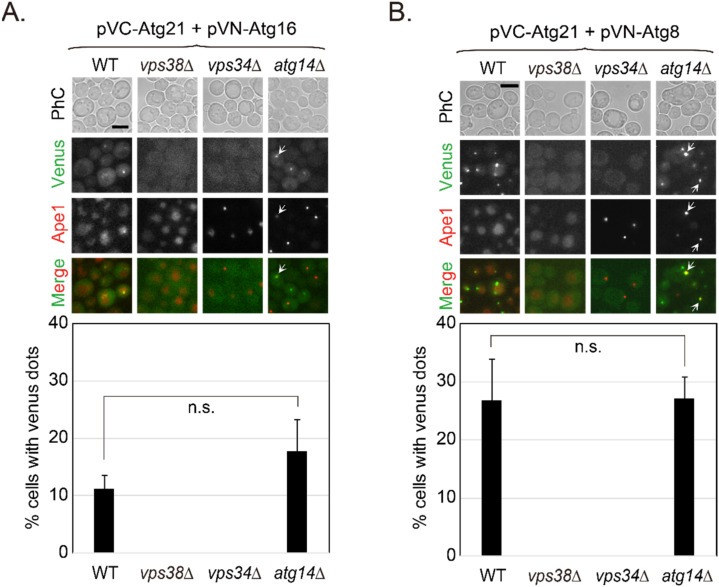
Atg21-Atg16 and Atg21-Atg8 interactions are dependent on PI3K complex II. (**A**) The Atg21-Atg16 interaction depended on Vps38 and Vps34 but not on Atg14. (**B**) The Atg21-Atg8 interaction depended on Vps38 and Vps34 but not on Atg14. The interactions were determined in BiFC assays using the indicated strains expressing RFP-Ape1 and BiFC plasmids. Arrows indicate the colocalization between Venus and Ape1 dots. The percentages of cells with Venus-positive dots were quantified and are shown below the merge pictures. Scale bars, 5 μm. n.s., not significant.

Vps21 regulates the localization of PI3K complexes, especially PI3K complex II, which further regulates the localization of Atg21. If Vps21 regulates the interaction between Atg21 and Atg16 or Atg8 through PI3K complex II, then the absence of PI3K complex II subunits would result in lost interactions between Atg21 and Atg16 or Atg8. We tested this hypothesis using mutants of PI3K complex subunits to perform BiFC assays for Atg21 and Atg16. Our results showed that the 10–20% of cells with interaction between Atg21 and Atg16 required Vps38 and Vps34 but not Atg14 (Figure 6A). Similarly, the 20–30% of cells with interaction between Atg21 and Atg8 required Vps38 and Vps34 but not Atg14 (Figure 6B). These results do not contradict the roles of Atg14 in autophagy and Vps38 in trafficking, as no evidence suggests that these interactions should disappear in *atg14*∆ cells, but not in *vps38*∆ cells. These results support the possibility that Vps21 controls interactions between Atg21 and Atg16 or Atg8 through PI3K complex II but not PI3K complex I.

### 2.7. Vps21-Dependent Atg21 Localization Is Important for Atg5 and Atg16 Localization to the PAS

In WT cells, Atg21 is primarily localized to endosomes and secondarily to the PAS/APs ([26] and Figure 4). The endosomal localization of Atg21 was not disrupted in *atg8*∆ or *atg1*∆ cells (Figure S8A,C). However, Atg21 localization was Vps21-dependent as most Atg21 disappeared from endosomes or the PAS in either *vps21*∆ or *vps21*∆*atg8*∆ cells (Figure S8). Atg21 is required for Atg16 localization to the PAS [18]. To examine the effect of the absence of Vps21 on the Atg12-Atg5·Atg16 complex, we constructed separate plasmids for Atg12, Atg5, and Atg16 and confirmed the expression and functions of these plasmids. The Atg12 plasmid complemented mCherry-Atg8 defects in *atg12*∆ cells (Figure S9D). Immunoblotting assays further confirmed the complementary effect of Atg12 on the Ape1 maturation defect in *atg12*∆ cells (Figure S9E). The Atg16 plasmid complemented the RFP-Ape1 defect in *atg16*∆ cells (Figure S9F). The Atg5 plasmid complemented the RFP-Ape1 defect in *atg5*∆ cells (Figure S9G,H). As showed in Figure S9I, all proteins encoded in the constructed plasmids were expressed properly, with no difference found in WT and *vps21*∆ cells.

**Figure 7:**
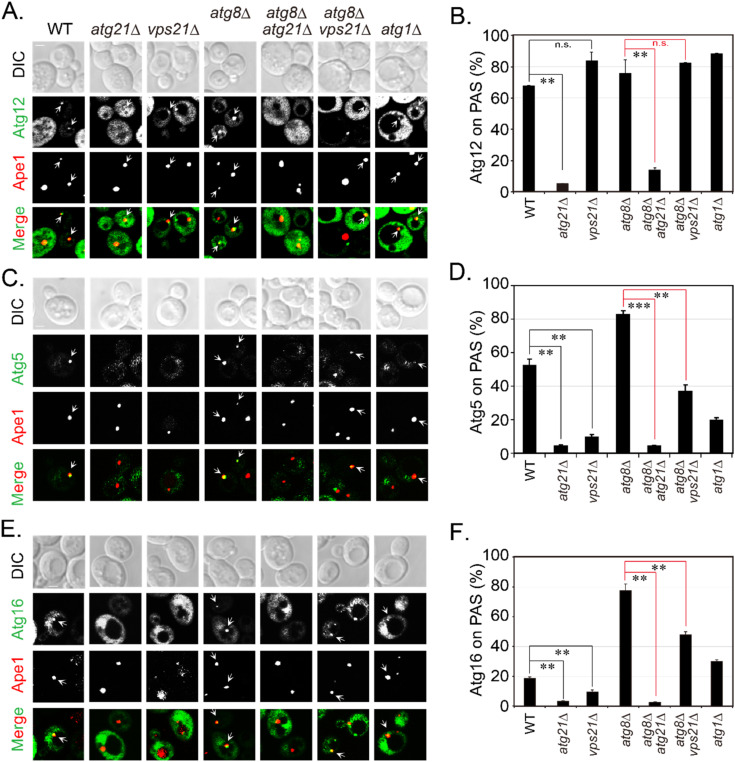
Localizations of Atg5 and Atg16 to RFP-Ape1-labeled PAS decrease in *vps21*∆ cells. The indicated strains were transformed with the pRS415-*CUP1p*-yEGFP-Atg12, pRS415-*CUP1p*-yEGFP-Atg16, and pRS415-*CUP1p*-Atg5-yEGFP plasmids. Cells were grown to mid-log phase, starved in SD-N medium for 2 h, and examined for fluorescence, as described in Figure 1D. (**A**) Atg12 localization to RFP-Ape1-labeled PAS/APs depended on Atg21 but not on Vps21. (**B**) Quantification of the data presented in panel (**A**). (**C**) Atg5 localization to RFP-Ape1-labeled PAS/APs depended on Atg21 and Vps21. (**D**) Quantification of the data presented in panel (**C**). (**E**) Atg16 localization to RFP-Ape1-labeled PAS/APs depended on Atg21 and Vps21. Scale bars, 5 μm. (**F**) Quantification of the data presented in panel (**E**). Arrows in panels (**A**,**C**,**E**) indicate the colocalization between green Atg and red Ape1 dots. The percentages of colocalized RFP-Ape1-labeled PAS/AP dots shown in panels (**A**,**C**,**E**) were quantified and presented in panels (**B**,**D**,**F**), respectively, as the mean ± SD. n.s., not significant; **, *p* < 0.01; ***, *p* < 0.001.

Since Vps21 regulated the localization of Atg21, we examined the localizations of Atg12, Atg5, and Atg16 to RFP-Ape1-labeled PAS in different mutants. Over 60% of Atg12 localization to the PAS in WT cells did not change markedly in cells lacking Vps21, although Atg12 was diffused in cells without Atg21 (Figure 7A,B). Further deletion of *ATG8* did not significantly change Atg12 localization to the PAS. Atg16 is a component of the Atg12-Atg5·Atg16 conjugation system [29], and Atg21 is also required for Atg5 localization to the PAS [18]. Over 40% of Atg5 and over 10% of Atg16 localized to the PAS in WT cells. However, Atg5 and Atg16 localizations to the PAS significantly decreased to less than 10% in cells lacking Vps21 or Atg21 (Figure 7C–F). These results indicate that Vps21 was partly required for Atg5 and Atg16 localizations to the PAS/APs, probably through its regulation by Atg21. These results are consistent with previous findings, wherein Atg16 mislocalized from the PAS in *atg21*∆ or *atg8*∆*atg21*∆ cells [30]. The decreased Atg5 and Atg16 localizations to the PAS in *vps21*∆ cells would be expected to contribute to autophagy defects in the cells. Strikingly, the localizations of Atg5 and Atg16 on the PAS significantly increased in *atg8*∆ cells in terms of both frequency and magnitude (fluorescence levels), but this change was not so prevalent in *atg1*∆ cells, suggesting that Atg8 competed with Atg5 and Atg16 in binding Atg21 on the PAS [18].

### 2.8. Vps21 and Atg21 Genetically Interact to Regulate Autophagy under Nitrogen Starvation

**Figure 8:**
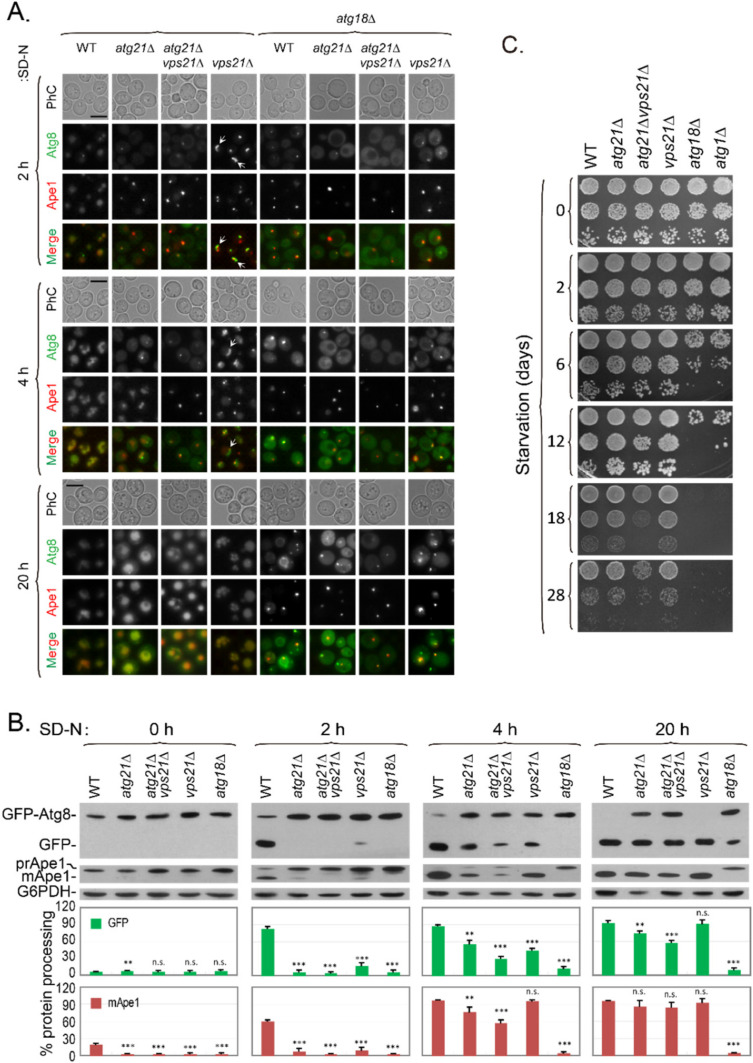
Vps21 and Atg21 synergistically regulate autophagy under nitrogen starvation. (**A**) GFP-Atg8 and RFP-Ape1 localizations were synergistically regulated by Atg21 and Vps21 under nitrogen-starvation conditions. The indicated strains were grown in YPD medium to mid-log phase and starved in SD-N medium for different durations, and GFP-Atg8 and RFP-Ape1 localizations were then observed. Strains with an *atg18*∆ background were used as controls for the *atg21*∆ strains. The arrows indicate autophagosomal clusters (APCs). Scale bars, 5 μm. (**B**) Immunoblotting assays indicated that GFP-Atg8 degradation and prApe1 maturation were synergistically regulated by Atg21 and Vps21 under nitrogen-starvation conditions. Anti-GFP and anti-Ape1 antibodies were used to assess GFP-Atg8 degradation and prApe1 maturation, respectively. G6PDH served as a loading control. The immunoblotting bands were quantified, and the mean ± SD are presented graphically below the bands. Significant differences are indicated with *p*-values in comparison to the WT strain: n.s., not significant; **, *p* < 0.01; ***, *p* < 0.001. (**C**) Growth assays for strains after different durations of nitrogen starvation indicated that Atg21 and Vps21 synergistically regulated autophagy. The main strains in panel (**A**), as well as an additional *atg1*∆ strain, were grown in YPD medium to mid-log phase and then starved in SD-N medium for different durations. The starved cells were washed with sterilized water, spotted onto YPD plates, and grown for 3 days. The results shown are representative of those from least two independent experiments.

To explore the physiological relevance of Vps21 and Atg21 in nitrogen starvation- induced autophagy, we determined autophagic defects in several strains over different durations. Atg21 was first reported to be only required for cytoplasm-to-vacuole targeting pathway [31]. However, a few subsequent reports showed that starvation-induced autophagy was also partially reduced in the absence of Atg21 [30,32,33]. We found that Atg21 was also required for nitrogen starvation-induced autophagy, although the defects decreased with longer starvation periods (Figure 8). Consistent with our previous report [20], this study revealed that Atg8 accumulated as clusters in *vps21*∆ cells. These clusters decreased as the starvation time increased (Figure 8A). Immunoblotting analysis showed that the autophagic defect in *vps21*∆ cells was slightly weaker than that in *atg21*∆ cells under the same conditions, as a higher percentage (about 15%) of GFP was observed in *vps21*∆ cells than in *atg21*∆ cells grown in SD-N for 2 h (Figure 8B). Importantly, the autophagic defects in double-mutant *vps21*∆*atg21*∆ cells were stronger than either of the *vps21*∆ or *atg21*∆ single-mutant cells grown in SD-N for 4 h (Figure 8A,B). We also found that *vps21*∆*atg21*∆ cells were less capable of resisting nitrogen starvation than the *vps21*∆ or *atg21*∆ cells (Figure 8C). These results indicate that Vps21 and Atg21 genetically interacted to regulate autophagy.

Atg21 and Atg18 are homologous yeast proteins that have redundant and diverse functions in autophagy and are often studied together [26], and the absence of Atg18 resulted in severe defects [30]. We included Atg18 as a control for Atg21 and found that the role of Atg18 in autophagy was more critical as a deletion of *ATG18* alone or together with *ATG21* and/or *VPS21* resulted in the complete blockage of nitrogen starvation-induced autophagy for up to 4 h, with GFP-Atg8 being present in the cytosol and Ape1 located in dots (Figure 8A). Our immunoblotting analysis results were consistent with our fluorescence observations (Figure 8B).

## 3. Discussion

**Figure 9:**
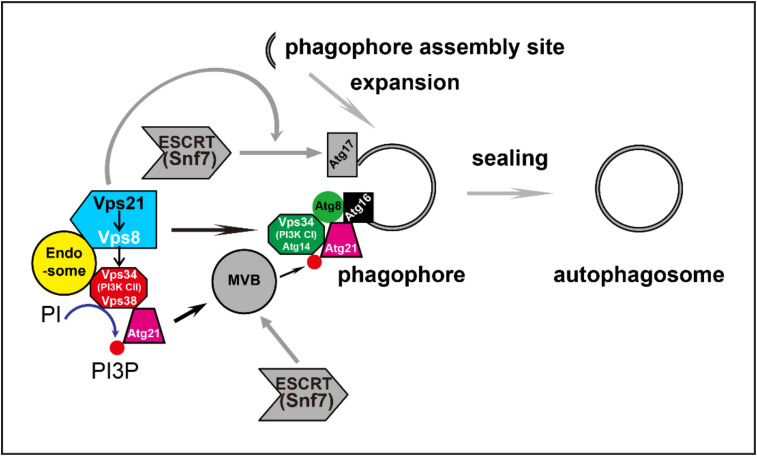
Proposed model of the roles of Vps21 in autophagy through regulating PI3K complexes and subsequent interactions. Vps21 controls the interaction between Vps8 and Vps34 and their localizations to endosomes. Vps21 further regulates the subsequent interactions between Vps34 and Atg21, Atg21 and Atg16, and Atg21 and Atg8 and the locations of these interactions. Defects in these localizations and/or interactions might impair the roles of Vps21 in progressing autophagy in the early stage. In addition, during the late stage, Vps21 regulates autophagosome closure by recruiting ESCRT via Snf7-Atg17 interaction [22,34]. Arrows and graphic symbols in gray color indicate published results.

Autophagy is coordinated by multiple proteins and the same proteins or complex may be involved in the regulation of different autophagy stages. Previously, we reported that the yeast Rab5/Vps21 module was required for phagophore closure. However, in vesicle trafficking, the Rab5/Vps21 module is also important for the localization of Vps34 and its product, PI(3)P [16]. Vps34 and PI(3)P are essential for autophagy initiation [10]. In this study, we explored whether Vps21 depletion might alter autophagy by impairing Vps34 and PI(3)P under nitrogen starvation. Our results indicate that the Vps21 effector Vps8 interacted with Vps34 on endosomes under the control of Vps21. Subsequent interactions between Vps34 and Atg21, and between Atg21 and Atg8 or Atg16, on the PAS/APs also depended on Vps21. Furthermore, the localization of Atg5 and Atg16 on the PAS/APs depended on Vps21 and Atg21, whereas the absence of both Vps21 and Atg21 led to deteriorated autophagy. We propose that Vps21 regulates early autophagy steps by controlling localizations and interactions along the Vps34-PI(3)P-Atg21-Atg16 axis from endosomes to the PAS/APs, and that Vps21 also regulates the later autophagy step of phagophore closure through ESCRT to fulfill its roles in autophagy (Figure 9).

The yeast PI3K complex has been shown to exist in two forms. These include an Atg14-specific PI3K complex I and a Vps38-specific PI3K complex II, each sharing the common subunits Vps15, Vps34, and Vps30/Atg6 [10]. Although a fifth subunit (Atg38) belonging to PI3K complex I was further reported in both yeast and human cells [35,36]. Both the yeast and mammalian PI3K complex I function in AP biogenesis [37,38]. Vps34 is a kinase that generates PI(3)P [7,31]. Factors that regulate the activity of Vps34, including modification of Vps34 activity and complex composition, can indirectly regulate PI(3)P levels [39,40,41,42]. The interconversion of PI(3)P to different PIs by kinases and phosphatases can directly regulate PI(3)P levels. Notably, phosphoinositide 3-phosphatase negatively regulated autophagy during the early stage and positively during the late stage [43,44,45,46], probably because PI(3)P turnover has opposing effects during different stages of autophagy.

Undoubtedly, the localizations of Vps34 and PI(3)P are also important. Vps34 and PI(3)P need to be strictly regulated in a spatiotemporal manner to function effectively in autophagy; otherwise, the localization and function of their effectors will be disrupted. Different PI(3)P-binding proteins are found in different species. While Atg18, Atg21, and Hsv2 are PI(3)P-binding proteins in yeast cells, WIPI1-4, ALFY, and DFCP are PI(3)P-binding proteins in mammalian cells. Although opposing idea exists regarding homologs of specific PI(3)P-binding proteins between yeasts and mammalian cells [47,48], these PROPPIN proteins have two conserved lipid-binding sites [49,50]. Atg21 or WIPI2 is recruited to the PAS or omegasomes via PI(3)P, indicating that PI(3)P needs to locate properly at these membrane structures in advance through Vps34 complex localization or PI(3)P transfer. Atg21 or WIPI2 further recruits Atg16/ATG16L of the Atg5-Atg12·Atg16 complex [18,51]) and orchestrate Atg8/LC3 lipidation [18,52]. We found that Atg21 localization and its interaction with other Atg proteins at the PAS were impaired (Figure 1G, Figure 2, Figure 4, Figure 5, Figure 6 and Figure 7), although the protein levels were unchanged in *vps21*∆ cells (Figure 4C,D). Such impairments also occurred in other species when the level and localization of Vps34 or PI(3)P were altered [51,52,53,54]. Ca^2+^-flux modification did not affect PI(3)P generation but affected WIPI1 (Atg18) localization to autophagosomal membranes and induced autophagy [55]. Our results suggest that, in yeast, the localizations of PI(3)P and PI(3)P-binding proteins are highly regulated by Vps21 during autophagy.

The composition and function of PI3K complexes in different species are not completely resolved [56]. Originally, Vps38-specific PI3K complex II was thought to function only in endocytosis, and Atg14-specific PI3K complex I was thought to function only in autophagy. However, the PI3K complex II-PI(3)P-Vps27 axis was recently reported to function in microautophagy induction and nutrient-stress adaptation [57]. Studies have also shown that the PI3K complex without VPS38 and ATG14 is viable and can synthesize PI(3)P to function in autophagy [56,58]. Mammalian cells can produce APs through enigmatic noncanonical VPS34-independent pathways. The phenotypes observed after VPS34 inactivation were rescued by PI(5)P [59]. Dissecting the roles of the PI3K complex II in autophagy was challenging in this study, because of the simultaneous mislocalization of Vps34-PI(3)P-Atg21 to both endosomes and the PAS in yeast mutant cells. However, our results clearly indicate that not only did Vps34 and Atg21 mislocalize from both endosomes and the PAS in *vps21*∆ or *vps21*∆*atg8*∆ cells, but Atg14 also mislocalized from both endosomes and the PAS in *vps21*∆*atg8*∆ cells (Figure 3, Figure 4 and Figures S3–S6). These results indicate that Vps21 not only regulates the PI3K complex II on endosomes, but it also regulates the PI3K complex I and Atg21 at the PAS. Considering the further effects on Atg21 effectors and their interactions at the PAS, we propose that Vps21 also regulates the early autophagy stage. It is unclear how PI(3)P localized on the PAS to recruit Atg21, although one possibility is that lipids and Atg21 were transferred from endosomes to the PAS through Atg2 and Vps13 [60]. These uncertainties and possibilities need to be investigated in the future.

PI(3)P is well-known to be required for autophagy initiation, and its role in late autophagy cannot be excluded as the Atg14-Vps34 complex-generated PI(3)P is required for recruiting the Ypt7 module to APs in order to fulfill AP-vacuole fusion [61]. Several other proteins can function at multiple steps of autophagy through different molecular mechanisms. For example, APs fail to form in the Ykt6 mutant, indicating that Ykt6 plays a role in early autophagy [62]. Meanwhile, Ykt6 on APs is also required for AP-vacuole fusion [61]. Another example demonstrates that the Atg17-Atg31-Atg29 complex coordinates with Atg11 to recruit the Vam7 SNARE to mediate AP-vacuole fusion beyond the essential role of the complex in autophagy induction [63]. Our previous report showed that Vps21 regulates a late step of autophagy by controlling the role of ESCRT in sealing phagophores [21,22,34], and here, we reported that Vps21 functions in early autophagy by controlling the production of PI(3)P to regulate the localization of Atg to the PAS.

A limitation of this study is that we did not determine the mechanism underlying the additive defect in autophagy when Vps21 and Atg21 were both depleted. In the future, a detailed analysis of our current observations might enhance the understanding of how the Vps21 module regulated autophagy. Furthermore, whether the Vps21 homolog Rab5 serves conserved functions in plant and animal cells in regulating autophagy through the PI3K complex and Atg21 is another important question that needs to be addressed.

In summary, we found that Vps21 not only regulates the Vps34-PI(3)P-Atg21 axis on endosomes but also regulates the localizations of Atg21 and Atg14 and the interactions between Atg21 and Atg16 or Atg8 on the PAS/APs. These results shed light on the functions of Vps21-module proteins on autophagy at the early stage, as well as their regulatory roles in phagophore closure by recruiting the ESCRT complex through Atg17-Snf7 interactions, which promotes phagophore sealing at the late stage [21,22,34]. However, it is presently unclear how these processes are coordinated.

## 4. Materials and Methods

### 4.1. Strains, Plasmids, and Reagents

The yeast strains and plasmids used in this study are listed in Table S1. All yeast and *Escherichia coli* transformations were performed as previously described [64].

The chromosomal sequences of *VPS8*, *VPS34*, *VPS38*, *ATG14*, and *ATG21* were modified in different background strains through polymerase chain reaction (PCR) amplification and recombination techniques to tag mNeonGreen or tdTomato at the C-terminus. Atg12, Atg5, and Atg16 expression plasmids were constructed using the pRS415-*CUP1p*-yEGFP vector and their functions were verified via fluorescence microscopy observations or immunoblotting assays before they were transformed into RFP-Ape1-labeled strains. For triple-colocalization analysis, a Snf7-2xmTagBFP2 plasmid was linearized using Afe1 and integrated into the above strains that already expressed two proteins with different fluorescent tags. While generating a mutant, the open reading frame of the target gene in the WT strain was replaced with a drug-resistance cassette (*hphMX4* or *kanMX3*) or the *LYS2* gene. This was achieved by using PCR amplification and recombination techniques. A DsRed-FYVE plasmid was used to probe PI(3)P in strains expressing Vps34-mNeonGreen. All colocalizations were examined under normal growth and autophagy induction conditions.

Plasmids were constructed as follows: The pRS415-*CUP1p*-yEGFP-Atg12 and the pRS415-*CUP1p*-yEGFP-Atg16 plasmids were constructed by cloning *ATG12* and *ATG16* into the pRS415-*CUP1p*-yEGFP vector, respectively. *ATG12* and *ATG16* were amplified with PrimerSTAR HS DNA Polymerase to obtain the PCR products to be used for cloning. The vector and PCR products were digested with *Sma*I and *Sac*II before being ligated with T4 DNA ligase to yield the pRS415-*CUP1p*-yEGFP-Atg12 and pRS415-*CUP1p*-yEGFP-Atg16 plasmids, respectively.

We failed to obtain a functional pRS415-*CUP1p*-yEGFP-Atg5 plasmid with this method. Therefore, DNA encoding yEGFP was inserted at the 3′-end of *ATG5* to construct the plasmid. Fragments encoding *CUP1p*, *ATG5* and yEGFP were amplified separately using PCR with corresponding primers. The three amplified DNA fragments were purified using an agarose gel extraction kit and used as templates to amplify *CUP1p*-Atg5-yEGFP. Then, the PCR product and pRS415 were digested with *Sma*I and *Sac*II and ligated with T4 DNA ligase to yield the pRS415-*CUP1p*-Atg5-yEGFP plasmid.

The Snf7-2xmTagBFP2-*TRP1* plasmid was constructed as follows. First, the 2xmTagBFP2 fragment was PCR-amplified from the ClhN-*SEC63*-2xmTagBFP2-*TRP1* plasmid. Then, the ClhN-*SNF7*-mCherry-*TRP1* plasmid was digested with *Nhe*I and *Avr*II to release the mCherry fragment. The digested products were separated by agarose gel electrophoresis, and the large fragment was extracted using an agarose gel extraction kit. This purified large fragment and the 2xmTagBFP2 fragment were ligated using a one-step cloning kit to yield the ClhN-*SNF7*-2xmTagBFP2-*TRP1* plasmid.

All antibodies and chemical reagents used in this study have been previously described [20,65].

### 4.2. Yeast Culture Conditions and Induction of Autophagy

Strains without plasmids were grown in YPD medium to mid-log phase and switched to SD-N medium for different durations, as previously described [20]. For complementation analyses with low-copy plasmids, mCherry-Atg8 or RFP-Ape1 tagged WT and *atgX*∆ cells were transformed with an empty vector (pRS415-*CUP1p*-yEGFP, 2µ, *LEU2*) or an AtgX-expressing plasmid. The transformants were grown in SD-Leu medium overnight and then starved in SD-N medium (0.17% yeast nitrogen base without amino acid and ammonium sulfate with 2% glucose) for 2 h at 26 °C, as previously described [66]. For complementation analysis, the ClhN-*SNF7*-2xmTagBFP2-*TRP1* plasmid was integrated into GFP-Atg8 tagged WT and *snf7*∆ cells, and the resulting cells were grown in SD-Trp medium overnight and then starved in SD-N medium for 2 h at 26 °C.

### 4.3. Fluorescence Microscopy Observations and Quantifications

Cells expressing fluorescently tagged proteins from plasmids and/or chromosomes, were examined using an Eclipse Ti inverted research microscope (Nikon, Tokyo, Japan) or an UltraVIEW spinning-disc confocal scanner unit (PerkinElmer, Waltham, MA, USA), as previously described [21]. More than five fields were visualized for each sample. In each experiment, the percentage of colocalized dots was quantified from 2 to 6 fields and the data are represented as the mean ± standard deviation (SD).

### 4.4. Y2H Assay

*VPS21* was deleted from the haploid genomes of Y2H host strains (Y2HGold and Y187) with a hygromycin-resistant gene cassette to generate *vps21*∆ cells. Then, the pACT2 or pGADT7 vector or gene-carrying plasmids were transformed into Y187 and Y187 *vps21*∆ cells. Correspondingly, the pGBKT7 vector or gene-carrying plasmids were transformed into Y2HGold and Y2HGold *vps21*∆ cells. Subsequently, the cells were mated on YPD medium and selected on SD-Trp-Leu plates to select diploid carrying opposite vectors and/or plasmids. The interactions were examined on SD-Trp-Leu-His plates at 26 °C after 2–3 days. Expression from the vectors and/or plasmids was examined using immunoblotting assays. The functions of the Y2H plasmids were verified by performing complementary assays. In some experiments, *ypt7*∆ cells were used as controls for *vps21*∆ cells.

### 4.5. BiFC Assay

The pVC and pVN vectors [67] were used to construct the pVC-Atg21, pVN-Atg16, and pVN-Atg8 plasmids, which were used for BiFC assays. The functions of the BiFC plasmids were verified with complementary assays before they were transformed into target strains containing mCherry-Atg8, RFP-Ape1, and/or Snf7-2xmTagBFP2. Cells cotransformed with the pVC and pVN plasmids were grown to mid-log phase in SD-Ura-His medium at 26 °C, and protein expression was induced by transferring the cells to SD-Ura-His-Met medium and growing them for 1.5 h. The cells were further starved in SD-N medium for 30 min and examined using an Eclipse Ti inverted research microscope (Nikon) or an UltraVIEW spinning-disc confocal scanner unit.

### 4.6. Immunoblotting Analysis

Crude lysates from cells expressing fluorescently tagged proteins or Y2H cells were subjected to immunoblotting to analyze protein expression levels or autophagy processing in at least two independent experiments, as previously described [68,69]. Blots were probed with an anti-GFP antibody to determine the levels of GFP-tagged Atg8, Atg12, Atg5, and Atg16 variants; an anti-mNeonGreen antibody to determine the levels of mNeonGreen-tagged Vps34, Vps38, and Atg21 variants; or an anti-Ape1 antibody to determine the levels of processed proteins in cell lysates. An anti-HA antibody was used to determine the expression levels of the pACT2 or pGADT7 plasmids, and an anti-myc antibody was used to determine the expression levels of the pGBKT7 plasmids. An anti-G6PDH antibody was used to determine the expression levels of G6PDH, as a loading control. Immunoblotting bands were quantified using the ImageJ software (National Institutes of Health, USA) in cases where quantification was necessary.

### 4.7. Statistical Analyses

The data are reported as the mean ± SD calculated from at least two independent experiments. The Student’s *t*-test was used to determine statistical significances. The p-value was used to demonstrate the significance, which was represented as: n.s., not significant; *, *p* < 0.05; **, *p* < 0.01; and ***, *p* < 0.001.

## Supplementary Materials

The following supporting information can be downloaded at: www.mdpi.com/article/10.3390/ijms23179550/s1. References [70,71,72,73,74,75] are cited in the supplementary materials.
