# Supplementary material for: Vps21 Directs the PI3K-PI(3)P-Atg21-Atg16 Module to Phagophores via Vps8 for Autophagy
Source: Int J Mol Sci. 2022 Aug 23;23(17):9550. doi: 10.3390/ijms23179550 (PMC9455592; doi:10.3390/ijms23179550)
Supplement: Supplementary file 1 [file ijms-23-09550-s001.zip › ijms-1879267-supplementary/R1-3-082022-Suppl-2003-plain text.pdf]

International Journal of
Molecular Sciences

Article

Vps21 Directs the PI3K-PI(3)P-Atg21-Atg16 Module to Phagophores via Vps8 for Autophagy

Lei Zhao [1,†], Weiming You [1,†], Dan Sun [1], Hui Xu [1], Xia You [1], Haiqian Xu [1], Zulin Wu [1], Zhiping Xie [2] and Yongheng Liang [1,*]

[1] College of Life Sciences, Key Laboratory of Agricultural Environmental Microbiology of Ministry of Agriculture, Nanjing Agricultural University, Nanjing 210095, China
[2] School of Life Sciences and Technology, Shanghai Jiao Tong University, Shanghai 200240, China
† These authors contributed equally to the work
* Correspondence: liangyh@njau.edu.cn

Supplementary Information

Figure S1–S9

Table S1. Yeast strains and plasmids used in this study

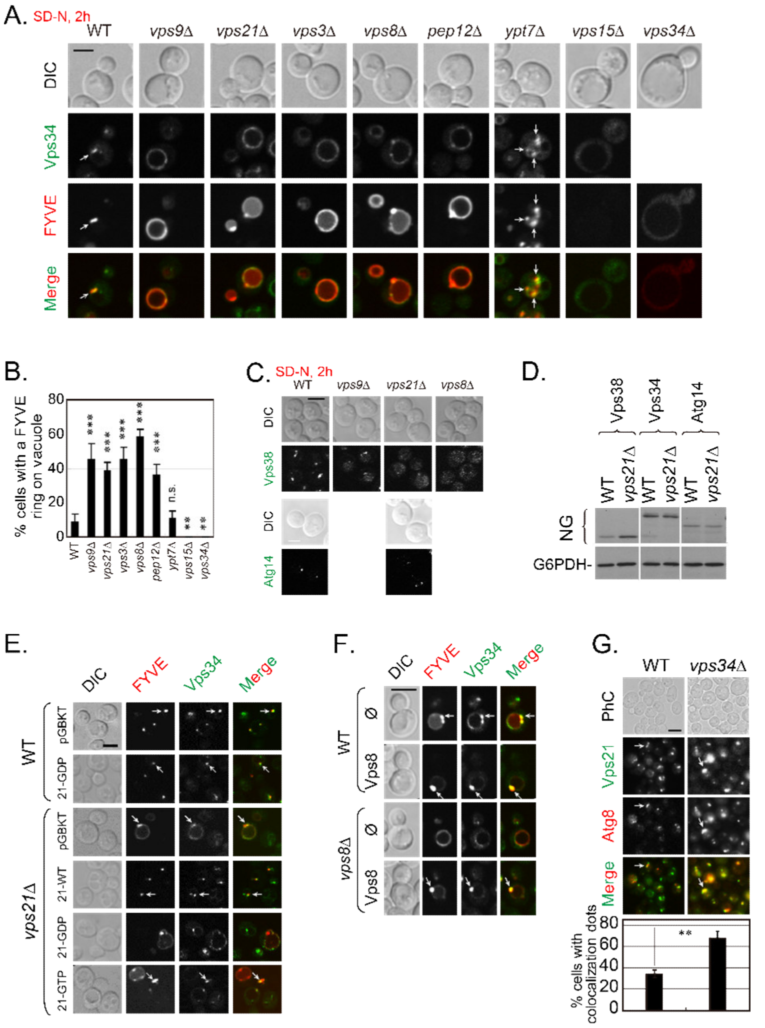

Figure S1. Representative subunits of PI3K complexes mislocalize in Vps21-module mutants under nitrogen starvation. **A**. Punctal colocalization of Vps34 and PI(3)P (probed with DsRed-FYVE) was impaired in Vps21 module mutants. Strains were grown in SD-Leu-Met to log phase, and then starved in SD-N medium for 2 h. The *vps15*Δ and *vps34*Δ strains were included as controls. **B**. Quantification of the percentage of cells with ring-like FYVE expression in vacuole membranes. Over 200 cells with FYVE expression as indicated at the bottom of panel A were quantified for each strain. **C**. Vps38 but not Atg14, showed diffusion in representative Vps21-module mutants. Strains expressing Vps38-mNeonGreen or Atg14-2XmNeonGreen were grown and treated as in panel A. **D**. The protein expression levels of Vps38, Vps34, and Atg14 were not decreased in *vps21*Δ cells. Cells were grown as described in panel C and subjected to immunoblotting analysis with an anti-mNeonGreen antibody. G6PDH served as a loading control. **E**. Normal localization of Vps34 and FYVE in *vps21*Δ cells was restored by WT-form of Vps21 and partially by GTP-form of Vps21. Cells were grown to log phase in SD-Trp and starved in SD-N medium for 2 h before being examined for fluorescence. **F**. Complementation of mislocalized Vps34 and FYVE in *vps8*Δ cells by cognate Vps8. The complementary ability of Vps8 was detected as described for panel E. **G**. Enhanced colocalization of Vps21 and Atg8 in *vps34*Δ cells. Cells were examined for fluorescence as described for panel A. The percentages of cells with Vps21-GFP and mCherry-Atg8 colocalization are presented as the mean ± SD below the merged pictures. The arrows point to examples of colocalization. Significant differences in B and G are indicated with p-values with comparison to WT: n.s., not significant; **, $p < 0.01$;◎***, $p < 0.001$. DIC, differential interference contrast; PhC, phase contrast. Scale bars, 5 µm.

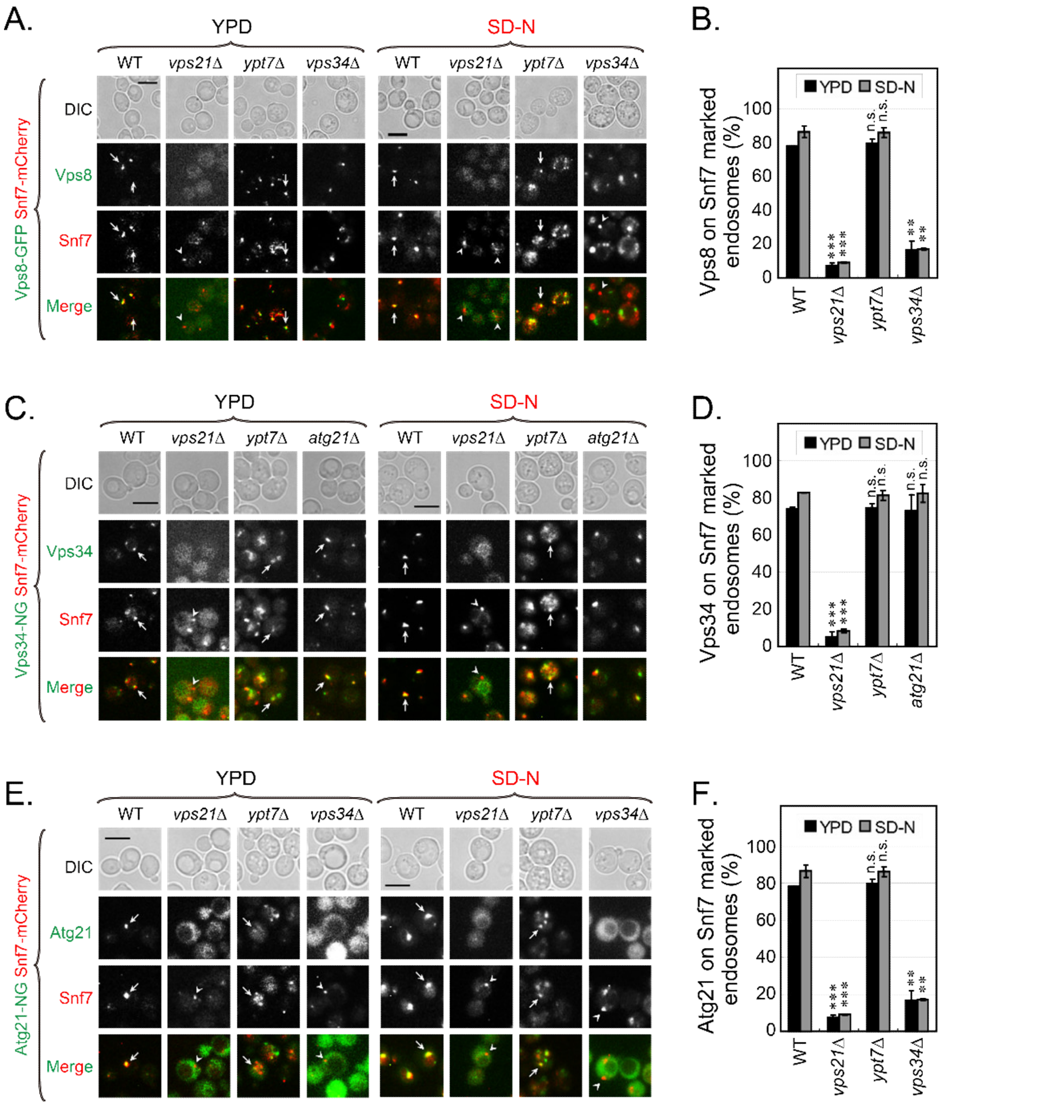

Figure S2. Like Vps34, Vps8 mislocalizes from endosomes in *vps21*Δ cells, and endosomal Vps34 localization is important for Atg21 recruitment to endosomes. A–B. Vps8 mislocalized from Snf7-mCherry-labeled endosomes in *vps21*Δ and *vps34*Δ cells in YPD (left) and SD-N (right) medium. Vps8-mNeonGreen and Snf7-mCherry-labeled cells were grown in rich medium to mid-log phase, or they were further starved in SD-N medium for 2 h before being examined for fluorescence. The percentages of dots showing colocalization were quantified and are presented as the mean ± SD. C–D. Vps34 mislocalized from Snf7-mCherry-labeled endosomes in *vps21*Δ cells, but not in *atg21*Δ cells in YPD or SD-N medium, as indicated. Vps34-mNeonGreen and Snf7-mCherry-labeled cells were grown and examined for fluorescence, as described for panel A. The percentages of dots showing colocalization were quantified and are presented as the mean ± SD. E–F. Atg21 mislocalization from Snf7-mCherry-labeled endosomes in *vps21*Δ and *vps34*Δ cells in YPD or SD-N medium, as indicated. Experiments were conducted as described for panel A, except that Vps8-mNeonGreen was replaced with Atg21-mNeonGreen. The percentages of dots showing colocalization were quantified and are presented as the mean ± SD. The data presented represent the results of two independent experiments. The arrows point to representative examples of colocalization, and the arrowheads indicate representative Snf7-mCherry-positive dots without green fluorescence. Scale bars, 5 μm. n.s., not significant; **, $p < 0.01$;◎***, $p < 0.001$.

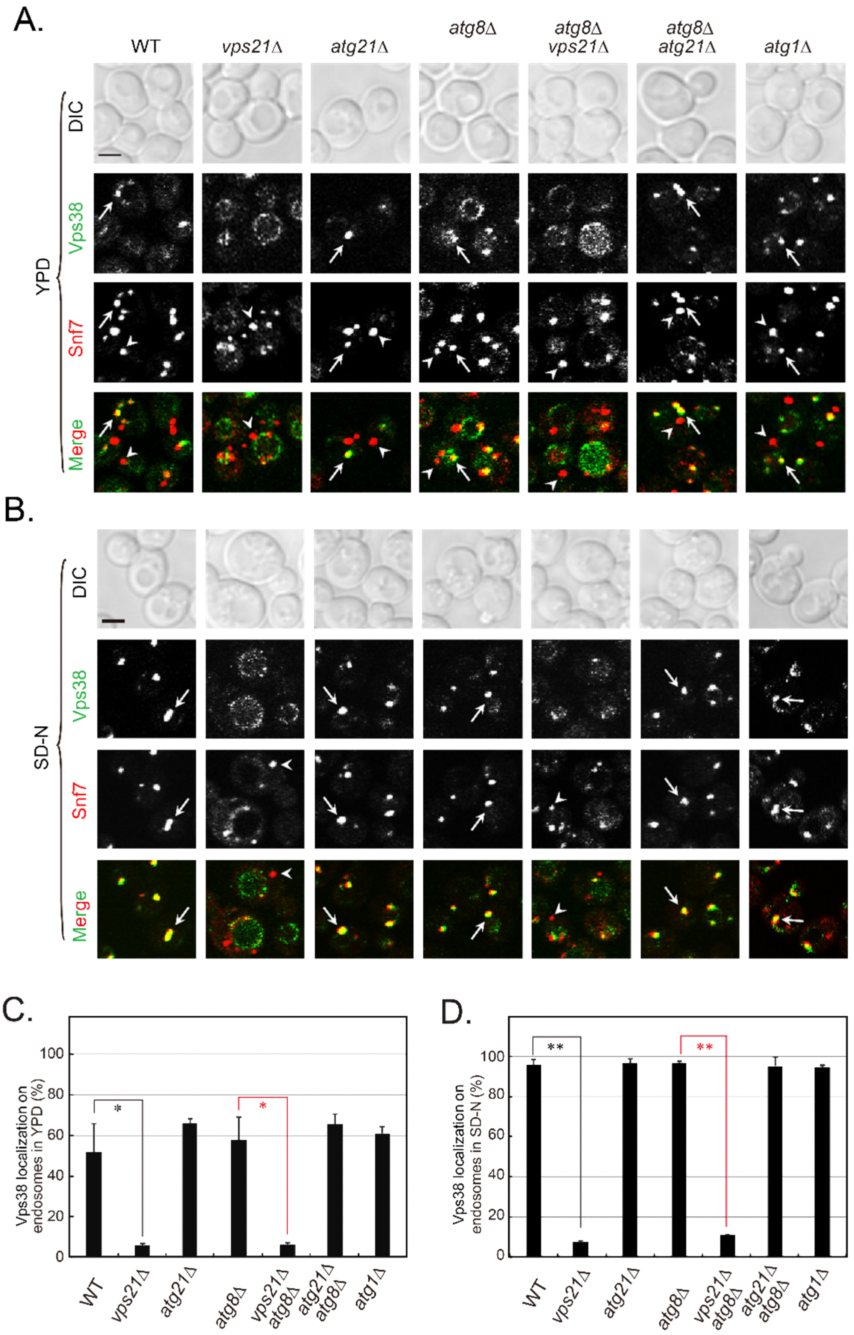

Figure S3. Endosomal Vps38 localization increases under nitrogen starvation but significantly decreases in *vps21Δ* cells under both rich and nitrogen-starvation conditions. A–B. Vps38-mNeonGreen mislocalized from Snf7-mCherry-labeled endosomes in *vps21Δ* and *vps21Δatg8Δ* cells but not in *atg21Δ* cells or other mutant cells. The indicated strains expressing Vps38-mNeonGreen and Snf7-mCherry were grown to mid-log phase in YPD (A) or further starved in SD-N medium for 2 h (B), after which fluorescence was examined. The arrows point to examples of colocalization, and the arrowheads indicate Snf7-mCherry-positive dots without Vps38-mNeonGreen expression. Scale bars, 2 μm. C–D. Quantification of Vps38 localization to Snf7-mCherry-labeled endosomes in different strains grown in YPD (C) or SD-N (D) medium. The percentages of dots showing colocalization dots are expressed as the mean ± SD. *,◎$p < 0.05$;◎**, $p < 0.01$.

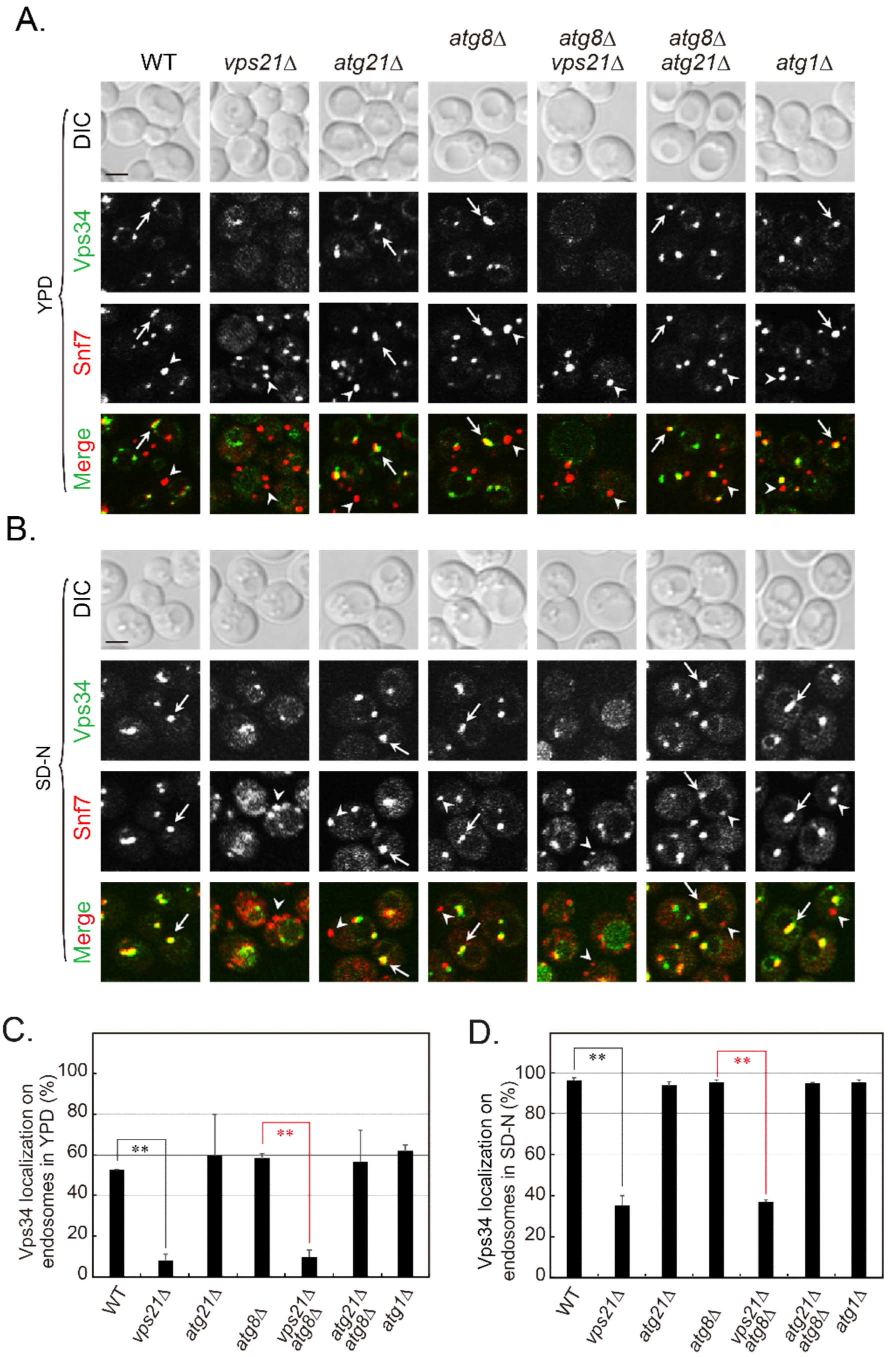

Figure S4. Endosomal localization of Vps34 increases under nitrogen starvation but significantly decreases in *vps21*Δ cells under both rich and nitrogen-starvation conditions. A–B. Vps34-mNeonGreen mislocalization from Snf7-mCherry-labeled endosomes in *vps21*Δ and *vps21*Δ*atg8*Δ cells but not in *atg21*Δ cells or other mutant cells. The indicated strains expressing Vps34-mNeonGreen and Snf7-mCherry were grown to mid-log phase in YPD (A) or further starved in SD-N medium for 2 h (B), after which fluorescence was examined. The arrows point to examples of colocalization, and the arrowheads indicate Snf7-mCherry-positive dots without Vps34-mNeonGreen expression. Scale bar, 2 µm. C–D. Quantification of Vps34 localization to Snf7-mCherry-labeled endosomes in different strains grown in YPD (C) or SD-N (D) medium. The percentages of dots showing colocalization are expressed as the mean ± SD. **, $p < 0.01$.

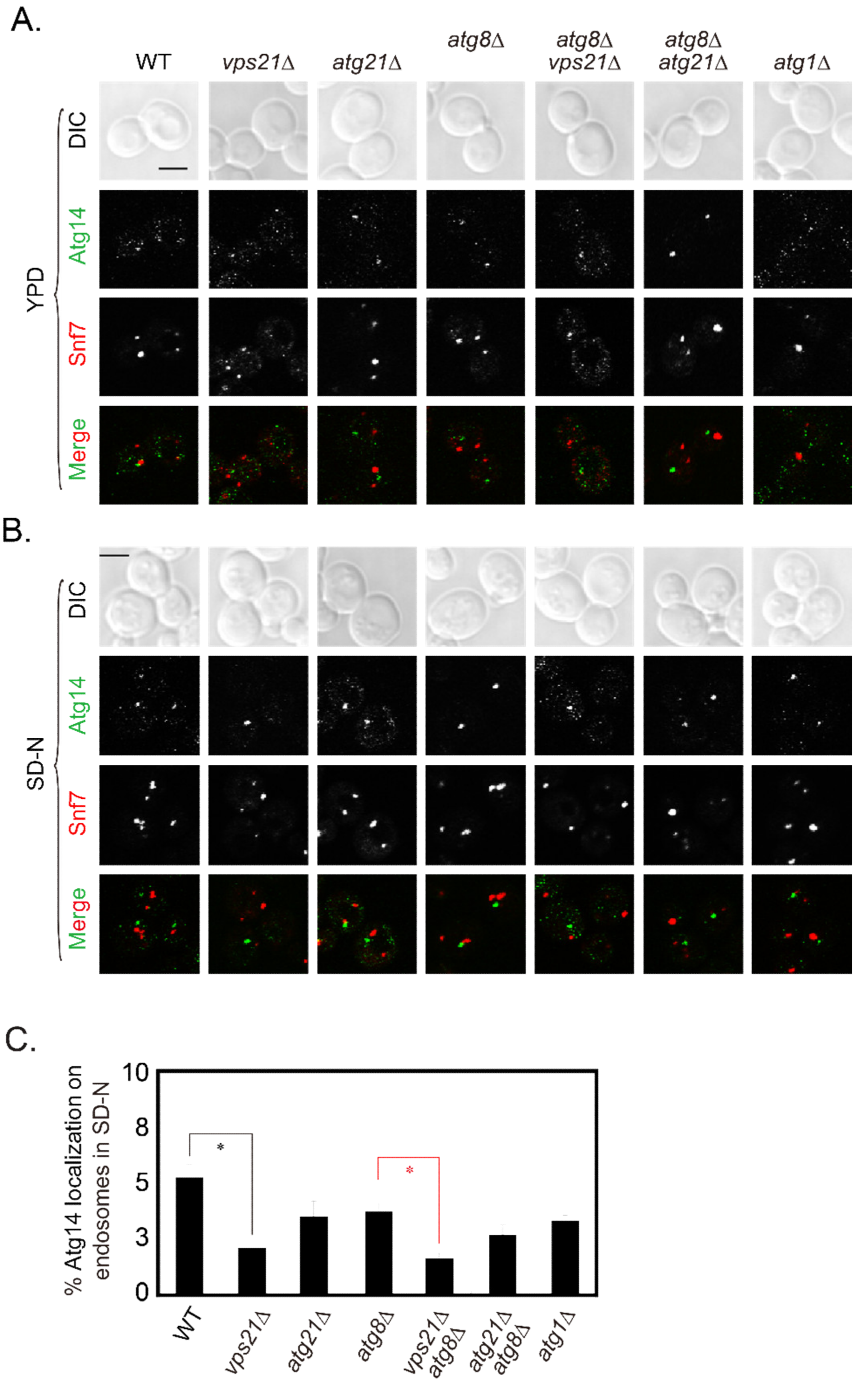

Figure S5. The scarce endosomal localization of Atg14 increases under nitrogen starvation but significantly decreases in *vps21Δ* cells under nitrogen starvation. A–B. The indicated strains expressing Atg14-2XmNeonGreen and Snf7-mCherry were grown to mid-log phase in YPD (A) or further starved in SD-N medium for 2 h (B), after which fluorescence was examined. Atg14-2XmNeonGreen mislocalized from Snf7-mCherry-labeled endosomes in *vps21Δ* and *vps21Δatg8Δ* cells. Scale bars, 2 μm. C. Quantification of Atg14-2XmNeonGreen localization to Snf7-mCherry-labeled endosomes in different strains grown in SD-N medium. The percentages of dots showing colocalization were expressed as the mean ± SD. *,⊛ $p < 0.05$.

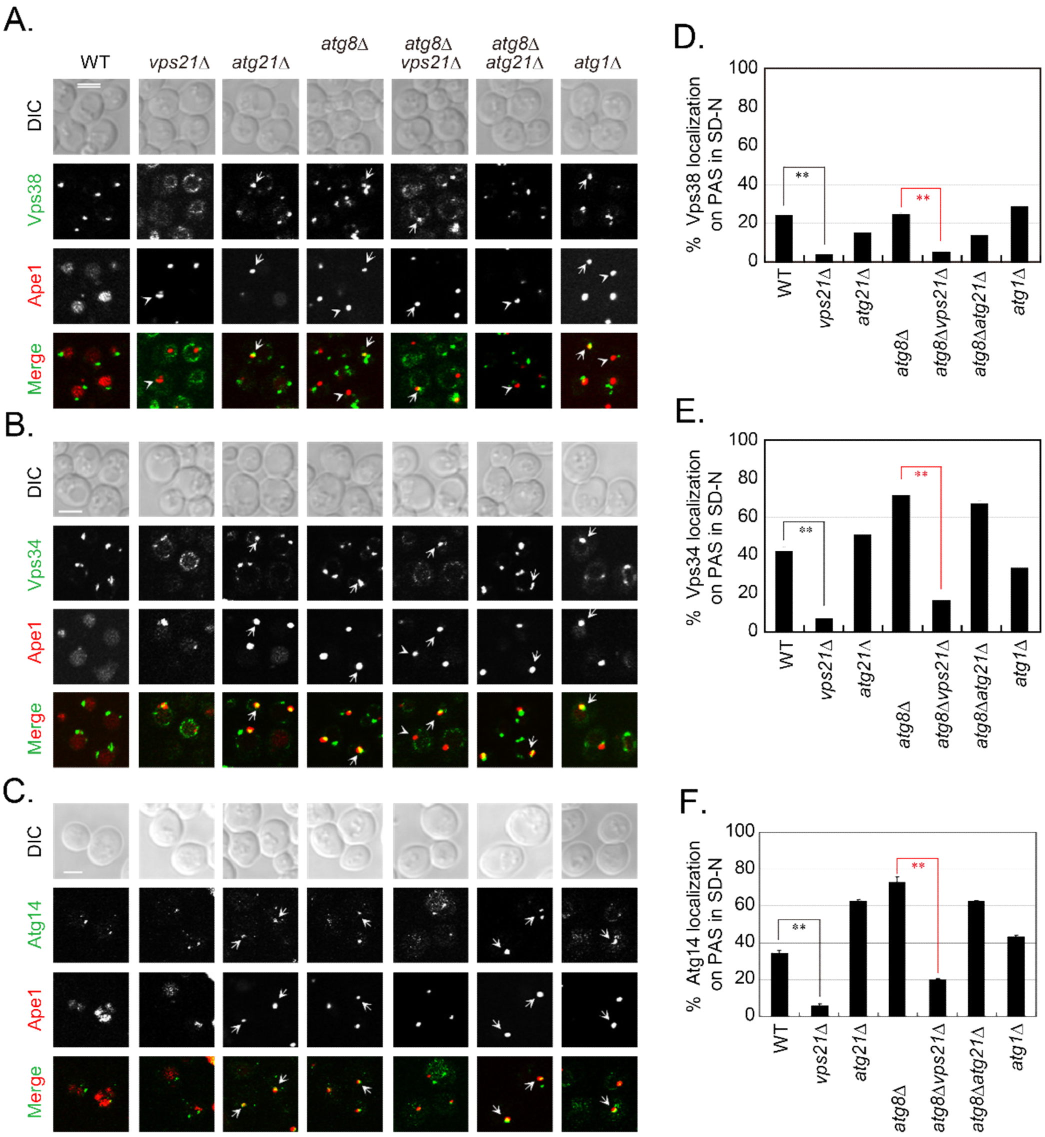

Figure S6. Localizations of representative PI3K subunits to RFP-Ape1-labeled PAS significantly decrease in *vps21Δ* cells under nitrogen starvation. A–C. Vps38-mNeonGreen (A), Vps34-mNeonGreen (B), or Atg14-2XmNeonGreen (C) mislocalized from RFP-Ape1-labeled PAS in *vps21Δ* cells. The indicated strains were grown to mid-log phase in YPD, further starved in SD-N medium for 2 h, after which fluorescence was examined. The arrows point to colocalization, and the arrowheads indicate RFP-Ape1-positive dots without green fluorescence. Scale bar, 2 μm. D–F. Quantification of Vps38-mNeonGreen (D), Vps34-mNeonGreen (E), or Atg14-2XmNeonGreen (F) localization to RFP-Ape1-labeled PAS in different strains grown in SD-N medium (represented in panels A–C). The percentages of dots showing colocalization were expressed as the mean ± SD. **, $p < 0.01$.

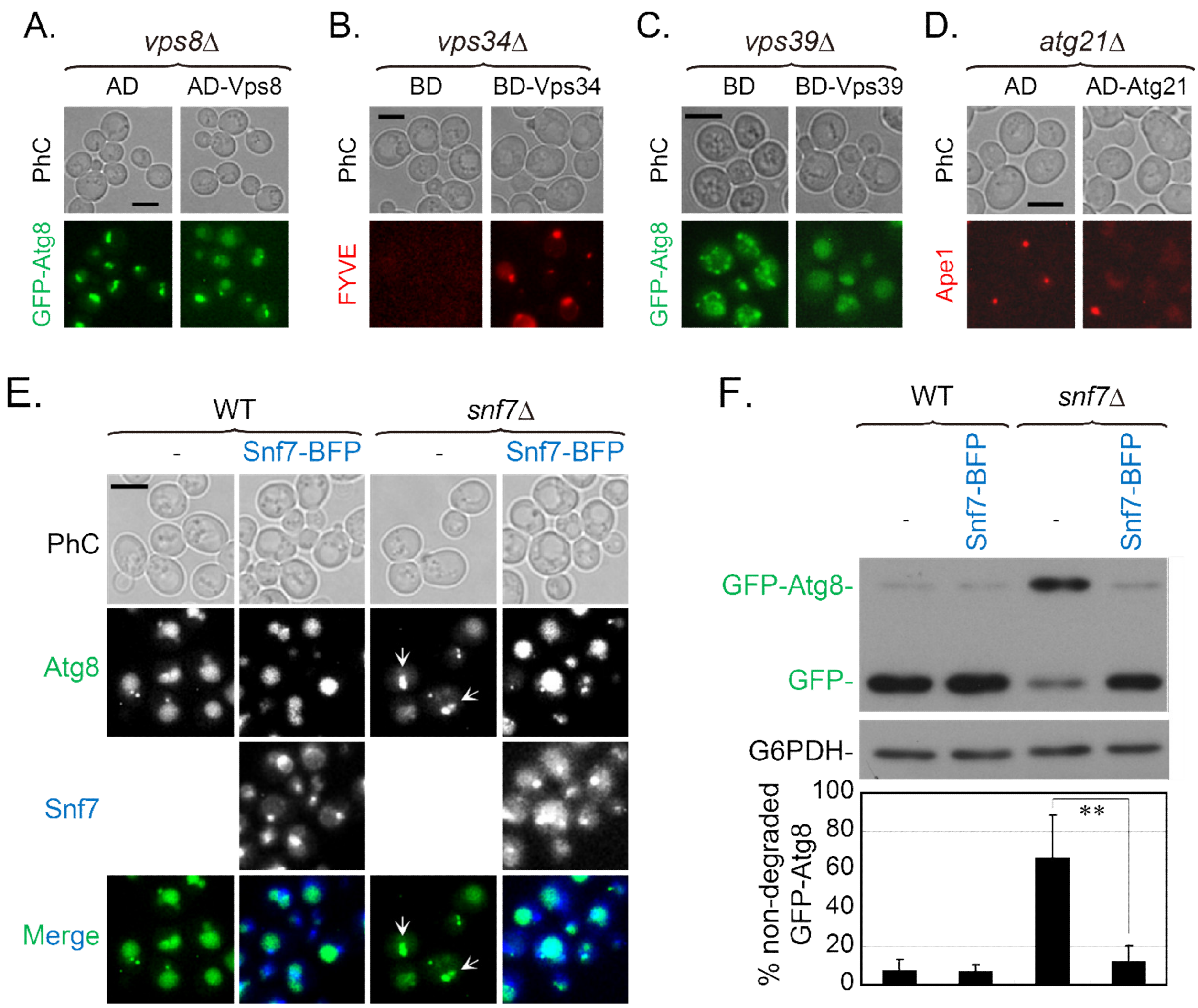

Figure S7. Y2H and Snf7-2xmTagBFP2 plasmids are functional. **A–D.** Fluorescence microscopy analysis indicated that the Y2H plasmids of pGADT7-Vps8 (A), pGBKT7-Vps34 (B), pGBKT7-Vps39 (C), and pGADT7-Atg21 (D) were functional. Mutant cells with an empty vector (left) or corresponding plasmid (right) were examined for fluorescence, as indicated. PhC, phase contrast. Scale bars, 5 µm. **E.** Fluorescence microscopy analysis indicated that the integrated Snf7-2xmTagBFP2 construct complemented the GFP-Atg8 accumulation phenotypes in *snf7*Δ cells. Snf7-2xmTagBFP2 was integrated into the chromosome of WT and *snf7*Δ cells expressing GFP-Atg8. The cells were grown in YPD, starved in SD-N medium for 2 h, and examined as described for Fig 3. The arrows point to APCs. Scale bars, 5 µm. **F.** Immunoblotting analysis indicated that the integrated Snf7-2xmTagBFP2 complemented autophagy defects in *snf7*Δ cells. Cells represented in panel E were examined and presented as in Fig 8B. The undegraded GFP-Atg8 bands were quantified and are presented below the blots as the mean ± SD. **, $p < 0.01$.

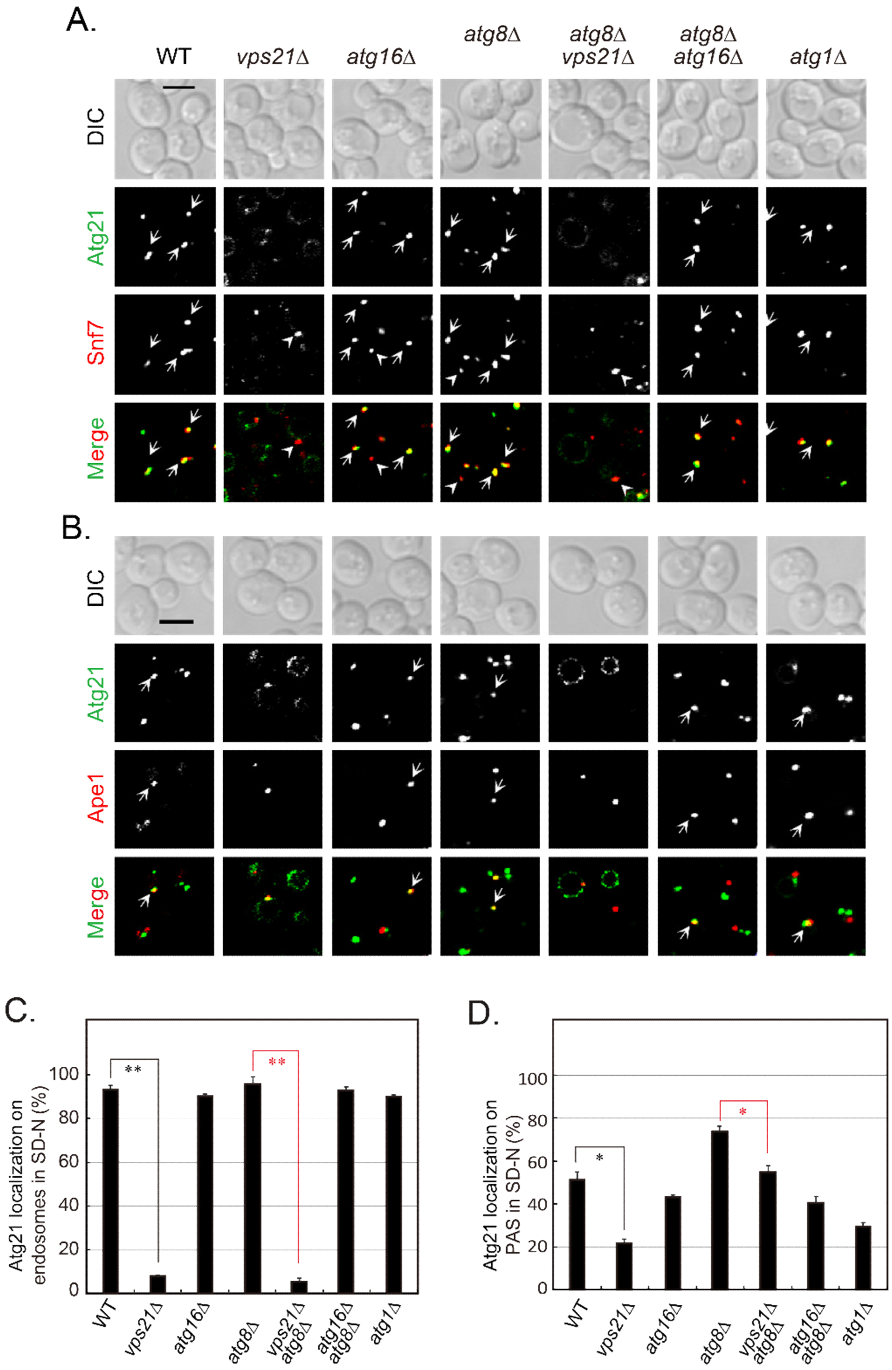

Figure S8. Localizations of Atg21 to endosomes and the PAS decrease significantly in *vps21Δ* cells under nitrogen starvation. A–B. The localization of Atg21-mNeonGreen to Snf7-mCherry-labeled endosomes (A) or to RFP-Ape1-labeled PAS (B) significantly decreased in *vps21Δ* and *vps21Δatg8Δ* cells. The experiments represented in this figure were performed as indicated in Fig S3B and Fig S6A, respectively. The arrows point to examples of colocalization, and the arrowheads indicate dots positive for Snf7-mCherry or RFP-Ape1 without Atg21-mNeonGreen. Scale bars, 5 μm. C–D. Quantification of Atg21 localization to Snf7-mCherry-labeled endosomes (D) or to RFP-Ape1-labeled PAS in different strains grown in SD-N medium. The percentages of dots showing colocalization dots are expressed as the mean ± SD. *,◎$p < 0.05$;◎**, $p < 0.01$. The results shown are representative of at least two independent experiments.

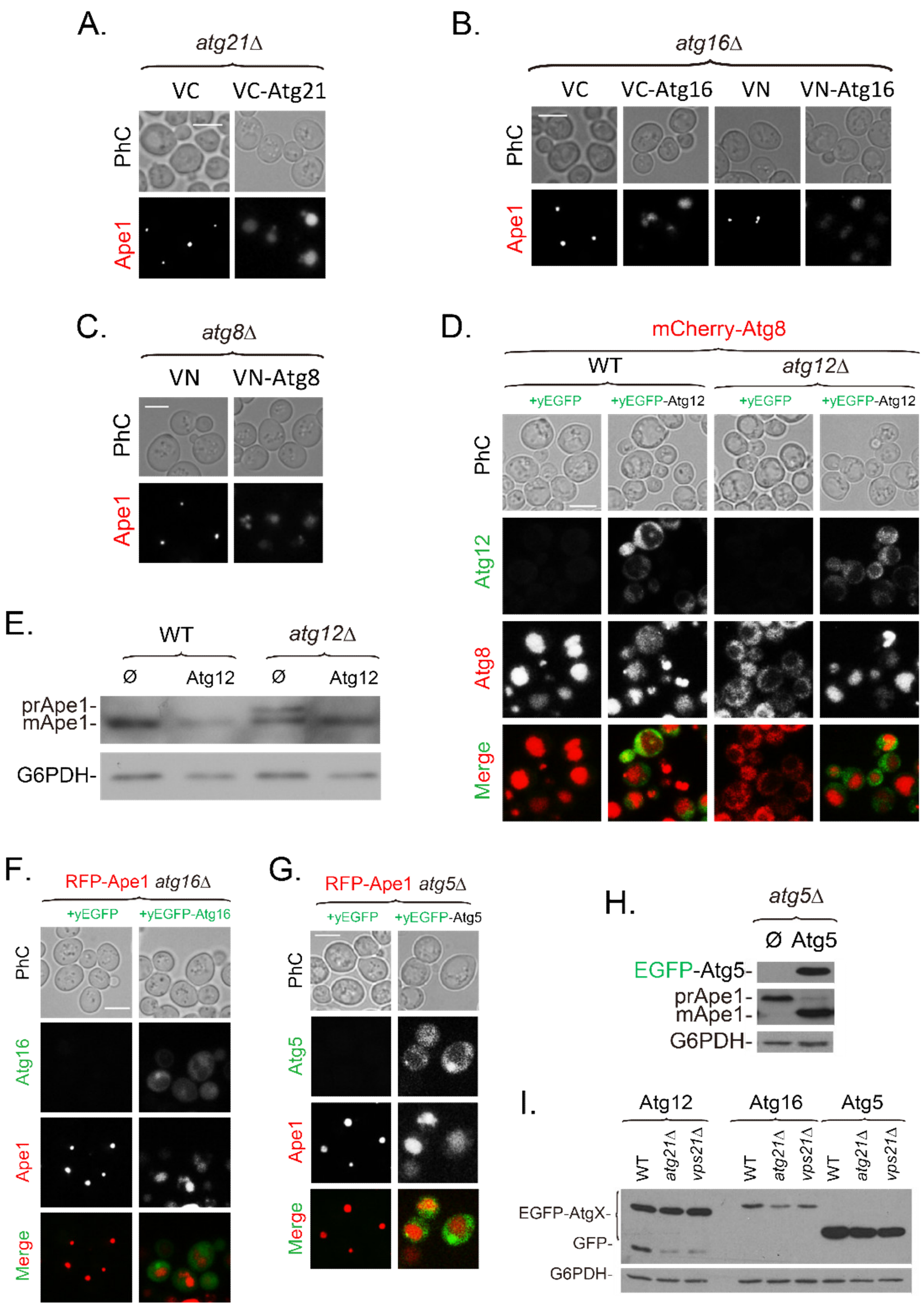

Figure S9. Functions of BiFC plasmids and the expression and functions of plasmids encoding Atg12-Atg5•Atg16-complex proteins. Cells grown to log phase were further starved in SD-N medium for 2 h before examination. A–C. The pVC-Atg21 (A), pVC-Atg16 and pVN-Atg16 plasmids (B), and pVN-Atg8 plasmids were functional. RFP-Ape1-expressing *atg21*Δ cells (A), *atg16*Δ cells (B), and *atg8*Δ cells transformed with indicated empty vector or plasmid were examined for RFP-Ape1 fluorescence as described for Fig 3. D. Fluorescence microscopy results indicating that the yEGFP-Atg12 plasmid was functional. The pRS415-*CUP1p*-yEGFP-Atg12 plasmid and the empty vector (pRS415-*CUP1p*-yEGFP) were used to transform mCherry-Atg8-expressing WT and *atg12*Δ cells. The cells were grown and examined for EGFP and mCherry fluorescence, as described for Fig 7. E. Immunoblotting assays showing that the yEGFP-Atg12 plasmid was functional. Defective Ape1 maturation in *atg12*Δ cells was complemented by pRS415-*CUP1p*-yEGFP-Atg12 and restored to similar levels seen in WT cells. The cells were grown as described for panel D, and Ape1 maturation was determined using an anti-Ape1 antibody. F–G. Fluorescence microscopy results indicating that yEGFP-Atg16 (F) and Atg5-yEGFP (G) were functional. The pRS415-*CUP1p*-yEGFP-Atg16, pRS415-*CUP1p*-Atg5-yEGFP plasmids and the empty vector (pRS415-*CUP1p*-yEGFP) were transformed into RFP-Ape1-expressing *atg16*Δ

cells (F) or *atg5*Δ cells (G) and examined as described for panel D. H. Immunoblotting assays showed that Atg5-yEGFP was expressed and functional. An anti-GFP antibody was used to detect Atg5-yEGFP and an anti-Ape1 antibody was used to detect Ape1 maturation, as described for E. I. No significant changes in the expression were observed for Atg12, Atg5, and Atg16 in *atg21*Δ and *vps21*Δ cells. The pRS415-*CUP1p*-yEGFP-Atg12, pRS415-*CUP1p*-yEGFP-Atg16, and pRS415-*CUP1p*-Atg5-yEGFP plasmids were transformed into WT, *atg21*Δ, and *vps21*Δ strains and treated as described for Fig 7. The expression levels of EGFP-Atg12, EGFP-Atg16, or Atg5-EGFP were examined with an anti-GFP antibody as described for panel H. PhC, phase contrast. Scale bar, 5 µm. In panels E and H–I, G6PDH served as a loading control. The results shown represent at least two independent experiments.

Table S1. Yeast strains and plasmids used in this study.

A. Strains

Strain	Genotype	Source	Figures
YLY3238	Y2H Gold	Clontech	Fig. 1A-B, 2C-D
YLY2569	Y187	Clontech	Fig. 1A-B, 2C-D
YLY5262	Y2HGoLd *vps21Δ::hphMX4*	This study	Fig. 1A-B, 2C-D
YLY5261	Y187 *vps21Δ::hphMX4*	This study	Fig. 1A-B, 2C-D
YLY8287	SEY6210 *VPS8-mNeonGreen-KanMX3 VPS38-tdTomato-TRP1*	This study	Fig. 1C
YLY8289	SEY6210 *VPS8-mNeonGreen-KanMX3 VPS34-tdTomato-TRP1*	This study	Fig. 1C
YLY8291	SEY6210 *VPS8-mNeonGreen-KanMX3 Atg14-tdTomato-TRP1*	This study	Fig. 1C
YLY10260	SEY6210 *SNF7-mCherry::TRP1*	This study	Fig. 1D
YLY3150	SEY6210 *SNF7-mCherry::TRP1 VPS38-VN::HisMX*	This study	Fig. 1D
YLY3151	SEY6210 *SNF7-mCherry::TRP1 VPS34-VN::HisMX*	This study	Fig. 1D
YLY3134	SEY6210 *SNF7-mCherry::TRP1 Atg14-VN::HisMX*	This study	Fig. 1D
YLY3152	SEY6210 *SNF7-mCherry::TRP1 VPS8-VC::KanMX3 VPS38-VN::HisMX*	This study	Fig. 1D
YLY10251	SEY6210 *SNF7-mCherry::TRP1 VPS8-VC::KanMX3 VPS34-VN::HisMX*	This study	Fig. 1D-E
YLY3149	SEY6210 *SNF7-mCherry::TRP1 VPS8-VC::KanMX3 Atg14-VN::HisMX*	This study	Fig. 1D
YLY10254	SEY6210 *SNF7-mCherry::TRP1 VPS8-VC::KanMX3 VPS34-VN::HisMX vps21Δ::hphMX4*	This study	Fig. 1E

YLY10253	SEY6210 *SNF7-mCherry::TRP1 VPS8-VC::KanMX3 VPS34-VN::HisMX atg1Δ::hphMX4*	This study	Fig. 1E
YLY9129	SEY6210 *ATG8::mCherry-ATG8-TRP1 VPS8-VC::KanMX3 VPS34-VN::HisMX*	This study	Fig. 1F
YLY9224	SEY6210 *ATG8::mCherry-ATG8-TRP1 VPS8-VC::KanMX3 VPS34-VN::HisMX atg21Δ::hphMX4*	This study	Fig. 1F
YLY9065	SEY6210 *Atg21-tdTomato-TRP1 VPS8-VC::KanMX3 VPS34-VN::HisMX*	This study	Fig. 1G
YLY9081	SEY6210 *Atg21-tdTomato-TRP1 VPS8-VC::KanMX3 VPS34-VN::HisMX vps21Δ::hphMX4*	This study	Fig. 1G
YLY9259	SEY6210 *Atg21-tdTomato-TRP1 VPS8-VC::KanMX3 VPS34-VN::HisMX atg1Δ::hphMX4*	This study	Fig. 1G
YLY8244	SEY6210 *Atg21-tdTomato-TRP1 VPS34-mNeonGreen-KanMX3*	This study	Fig. 2A-B
YLY8278	SEY6210 *Atg21-tdTomato-TRP1 VPS34-mNeonGreen-KanMX3 vps21Δ::hphMX4*	This study	Fig. 2A-B
YLY8417	SEY6210 *Atg21-tdTomato-TRP1 VPS34-mNeonGreen-KanMX3 ypt7Δ::hphMX4*	This study	Fig. 2A-B
YLY6074	Y187 *ypt7Δ::hphMX4*	This study	Fig. 2C-D
YLY6076	Y2H Gold *ypt7Δ::hphMX4*	This study	Fig. 2C-D
YLY10872	SEY6210 *VPS38-mNeonGreen-KanMX3 leu2::RFP-APE1-LEU2 SNF7-2xmTagBFP2::TRP*	This study	Fig. 3A, D
YLY10873	SEY6210 *VPS38-mNeonGreen-KanMX3 leu2::RFP-APE1-LEU2 SNF7-2xmTagBFP2::TRP vps21Δ::hphMX4*	This study	Fig. 3A, D
YLY10866	SEY6210 *VPS38-mNeonGreen-KanMX3 leu2::RFP-APE1-LEU2 SNF7-2xmTagBFP2::TRP atg8Δ::His3*	This study	Fig. 3A, D
YLY10867	SEY6210 *VPS38-mNeonGreen-KanMX3 leu2::RFP-APE1-LEU2 SNF7-2xmTagBFP2::TRP atg8Δ::His3 vps21Δ::hphMX4*	This study	Fig. 3A, D
YLY10868	SEY6210 *VPS34-mNeonGreen-KanMX3*	This study	Fig. 3B, D

	leu2::RFP-APE1-LEU2 SNF7-2xmTagBFP2::TRP		
YLY10869	SEY6210 *VPS34-mNeonGreen-KanMX3 leu2::RFP-APE1-LEU2 SNF7-2xmTagBFP2::TRP vps21Δ::hphMX4*	This study	Fig. 3 B, D
YLY10878	SEY6210 *VPS34-mNeonGreen-KanMX3 leu2::RFP-APE1-LEU2 SNF7-2xmTagBFP2::TRP atg8Δ::His3*	This study	Fig. 3 B, D
YLY10871	SEY6210 *VPS34-mNeonGreen-KanMX3 leu2::RFP-APE1-LEU2 SNF7-2xmTagBFP2::TRP atg8Δ::His3 vps21Δ::hphMX4*	This study	Fig. 3 B, D
YLY10848	SEY6210 *Atg14*-2x*mNeonGreen-KanMX3 leu2::RFP-APE1-LEU2 SNF7-2xmTagBFP2::TRP*	This study	Fig. 3C, D
YLY10849	SEY6210 *Atg14*-2x*mNeonGreen-KanMX3 leu2::RFP-APE1-LEU2 SNF7-2xmTagBFP2::TRP vps21Δ::hphMX4*	This study	Fig. 3C, D
YLY10850	SEY6210 *Atg14*-2x*mNeonGreen-KanMX3 leu2::RFP-APE1-LEU2 SNF7-2xmTagBFP2::TRP atg8Δ::His3*	This study	Fig. 3C, D
YLY10859	SEY6210 *Atg14*-2x*mNeonGreen-KanMX3 leu2::RFP-APE1-LEU2 SNF7-2xmTagBFP2::TRP atg8Δ::His3 vps21Δ::hphMX4*	This study	Fig. 3C, D
YLY10852	SEY6210 *Atg21-mNeonGreen-KanMX3 leu2::RFP-APE1-LEU2 SNF7-2xmTagBFP2::TRP*	This study	Fig. 4
YLY10861	SEY6210 *Atg21-mNeonGreen-KanMX3 leu2::RFP-APE1-LEU2 SNF7-2xmTagBFP2::TRP vps21Δ::hphMX4*	This study	Fig. 4
YLY10881	SEY6210 *Atg21-mNeonGreen-KanMX3 leu2::RFP-APE1-LEU2 SNF7-2xmTagBFP2::TRP atg8Δ::His3*	This study	Fig. 4
YLY10863	SEY6210 *Atg21-mNeonGreen-KanMX3 leu2::RFP-APE1-LEU2 SNF7-2xmTagBFP2::TRP atg8Δ::His3 vps21Δ::hphMX4*	This study	Fig. 4
YLY10831	SEY6210 *ATG8::mCherry-ATG8-TRP1 SNF7-2xmTagBFP2::Leu*	This study	Fig. 5A-C

YLY10832	SEY6210 *ATG8::mCherry-ATG8-TRP1 SNF7-2xmTagBFP2::Leu vps21Δ::hphMX4*	This study	Fig. 5A-C
YLY10833	SEY6210 *ATG8::mCherry-ATG8-TRP1 SNF7-2xmTagBFP2::Leu ypt7Δ::hphMX4*	This study	Fig. 5A-C
YLY1408	SEY6210 *leu2::RFP-APE1-LEU2*	[20]	Fig. 6
YLY2213	SEY6210 *leu2::RFP-APE1-LEU2 vps38Δ::hphMX4*	This study	Fig. 6
YLY7820	SEY6210 *leu2::RFP-APE1-LEU2 vps34Δ::hphMX4*	This study	Fig. 6
YLY2211	SEY6210 *leu2::RFP-APE1-LEU2 atg14Δ::hphMX4*	This study	Fig. 6
YLY1941	SEY6210 *trp1::RFP-APE1-TRP1*	This study	Fig. 7, S9I
YLY3459	SEY6210 *trp1::RFP-APE1-TRP1 atg21Δ::hphMX4*	This study	Fig. 7, S9I
YLY3458	SEY6210 *trp1::RFP-APE1-TRP1 vps21Δ::hphMX4*	This study	Fig. 7, S9I
YLY3929	SEY6210 *trp1::RFP-APE1-TRP1 atg8Δ::His3*	This study	Fig. 7
YLY3930	SEY6210 *trp1::RFP-APE1-TRP1 atg8Δ::His3 atg21Δ::hphMX4*	This study	Fig. 7
YLY3934	SEY6210 *trp1::RFP-APE1-TRP1 atg8Δ::His3 vps21Δ::hphMX4*	This study	Fig. 7
YLY3557	SEY6210 *trp1::RFP-APE1-TRP1 atg1Δ::hphMX4*	This study	Fig. 7
YLY7396	SEY6210 *his3::GFP-Atg8-HIS3 leu2::RFP-APE1-LEU2*	This study	Fig. 8
YLY9717	SEY6210 *his3::GFP-Atg8-HIS3 leu2::RFP-APE1-LEU2 atg21Δ::hphMX4*	This study	Fig. 8
YLY9554	SEY6210 *his3::GFP-Atg8-HIS3 leu2::RFP-APE1-LEU2 atg21Δ::hphMX4 vps21Δ::KanMX3*	This study	Fig. 8
YLY4281	SEY6210 *his3::GFP-Atg8-HIS3 leu2::RFP-APE1-LEU2 vps21Δ::KanMX3*	This study	Fig. 8
YLY9969	SEY6210 *his3::GFP-Atg8-HIS3 leu2::RFP-APE1-LEU2 atg18Δ::hphMX4*	This study	Fig. 8
YLY10031	SEY6210 *his3::GFP-Atg8-HIS3 leu2::RFP-APE1-LEU2 atg18Δ::hphMX4 atg21Δ::KanMX3*	This study	Fig. 8A
YLY10035	SEY6210 *his3::GFP-Atg8-HIS3 leu2::RFP-APE1-LEU2 atg18Δ::hphMX4 atg21Δ::KanMX3 vps21Δ::Lys*	This study	Fig. 8A

YLY10003	SEY6210 *his3::GFP-Atg8-HIS3 leu2::RFP-APE1-LEU2 atg18Δ::hphMX4 vps21Δ::Lys*	This study	Fig. 8A
YLY9583	SEY6210 *his3::GFP-Atg8-HIS3 leu2::RFP-APE1-LEU2 atg1Δ::hphMX4*	This study	Fig. 8C
YLY4899	SEY6210 *VPS34*-GFP::*URA*	This study	Fig. S1A-B, S1E-F
YLY8075	SEY6210 *VPS34*-GFP::*URA vps9Δ::hphMX4*	This study	Fig. S1A-B
YLY7201	SEY6210 *VPS34*-GFP::*URA vps21Δ::KanMX3*	This study	Fig. S1A-B, S1E
YLY7457	SEY6210 *VPS34*-GFP::*URA vps3Δ::hphMX4*	This study	Fig. S1A-B
YLY8076	SEY6210 *VPS34*-GFP::*URA vps8Δ::KanMX3*	This study	Fig. S1A-B, S1F
YLY7245	SEY6210 *VPS34*-GFP::*URA pep12Δ::hphMX4*	This study	Fig. S1A-B
YLY7458	SEY6210 *VPS34*-GFP::*URA ypt7Δ::hphMX4*	This study	Fig. S1A-B
YLY7992	SEY6210 *VPS34*-GFP::*URA vps15Δ::KanMX3*	This study	Fig. S1A-B
YLY7996	SEY6210 *VPS34*-GFP::*URA vps34Δ::hphMX4*	This study	Fig. S1A-B
YLY6330	SEY6210 *VPS38-mNeonGreen-KanMX3 ATG8*::*mCherry-ATG8-TRP1*	This study	Fig. S1C
YLY3518	SEY6210 *VPS38-mNeonGreen-KanMX3 ATG8*::*mCherry-ATG8-TRP1 vps9Δ::hphMX4*	This study	Fig. S1C
YLY3516	SEY6210 *VPS38-mNeonGreen-KanMX3 ATG8*::*mCherry-ATG8-TRP1 vps21Δ::hphMX4*	This study	Fig. S1C
YLY3517	SEY6210 *VPS38-mNeonGreen-KanMX3 ATG8*::*mCherry-ATG8-TRP1 vps8Δ::hphMX4*	This study	Fig. S1C
YLY2731	SEY6210 *Atg14*-2x*mNeonGreen-KanMX3 leu2::RFP-APE1-LEU2*	This study	Fig. S1C-D, S6C, F
YLY3109	SEY6210 *Atg14*-2x*mNeonGreen-KanMX3 leu2::RFP-APE1-LEU2 vps21Δ::hphMX4*	This study	Fig. S1C-D, S6C, F
YLY10493	SEY6210 *VPS38-mNeonGreen-KanMX3 leu2::RFP-APE1-LEU2*	This study	Fig. S1D, S6A, D
YLY10494	SEY6210 *VPS38-mNeonGreen-KanMX3 leu2::RFP-APE1-LEU2 vps21Δ::hphMX4*	This study	Fig. S1D, S6A, D
YLY10477	SEY6210 *VPS34-mNeonGreen-KanMX3 leu2::RFP-APE1-LEU2*	This study	Fig. S1D, S6B, E

YLY10543	SEY6210 *VPS34-mNeonGreen-KanMX3 leu2::RFP-APE1-LEU2 vps21Δ::His3*	This study	Fig. S1D
YLY8081	SEY6210 *VPS34-mNeonGreen-KanMX3*	This study	Fig. S1E-F
YLY8213	SEY6210 *VPS34-mNeonGreen-KanMX3 vps21Δ::hphMX4*	This study	Fig. S1E
YLY3465	SEY6210 *VPS21::URA3-PHO5pr-GFP trp1::mCherry-Atg8-TRP1*	This study	Fig. S1G
YLY4484	SEY6210 *VPS21::URA3-PHO5pr-GFP trp1::mCherry-Atg8-TRP1 vps34Δ::hphMX4*	This study	Fig. S1G
YLY11331	SEY6210 *VPS8-mNeonGreen-KanMX3 SNF7-mCherry-TRP1*	This study	Fig. S2A-B
YLY11332	SEY6210 *VPS8-mNeonGreen-KanMX3 SNF7-mCherry-TRP1 vps21Δ::hphMX4*	This study	Fig. S2A-B
YLY11333	SEY6210 *VPS8-mNeonGreen-KanMX3 trp1::SNF7-mCherry-TRP1 ypt7Δ::hphMX4*	This study	Fig. S2A-B
YLY11677	SEY6210 *VPS8-mNeonGreen-KanMX3 trp1::SNF7-mCherry-TRP1 vps34Δ::hphMX4*	This study	Fig. S2A-B
YLY11046	SEY6210 *VPS34-mNeonGreen-KanMX3 trp1::SNF7-mCherry-TRP*	This study	Fig. S2C-D, S4
YLY11047	SEY6210 *VPS34-mNeonGreen-KanMX3 SNF7-mCherry-TRP1 vps21Δ::hphMX4*	This study	Fig. S2C-D, S4
YLY2811	SEY6210 *VPS34-mNeonGreen-KanMX3 SNF7-mCherry-TRP1 ypt7Δ::hphMX4*	This study	Fig. S2C-D
YLY10597	SEY6210 *VPS34-mNeonGreen-KanMX3 SNF7-mCherry-TRP1 atg21Δ::hphMX4*	This study	Fig. S2C-D, S4
YLY10156	SEY6210 *Atg21-mNeonGreen-KanMX3 SNF7-mCherry-TRP1*	This study	Fig. S2E-F, S8A, C
YLY10326	SEY6210 *Atg21-mNeonGreen-KanMX3 SNF7-mCherry-TRP1 vps21Δ::Lys*	This study	Fig. S2E-F, S8A, C
YLY2902	SEY6210 *Atg21-mNeonGreen-KanMX3 SNF7-mCherry-TRP1 ypt7Δ::hphMX4*	This study	Fig. S2E-F
YLY2903	SEY6210 *Atg21-mNeonGreen-KanMX3 SNF7-mCherry-TRP1 vps34Δ::hphMX4*	This study	Fig. S2E-F

YLY10923	SEY6210 *VPS38-mNeonGreen-KanMX3 SNF7-mCherry-TRP1*	This study	Fig. S3
YLY10925	SEY6210 *VPS38-mNeonGreen-KanMX3 SNF7-mCherry-TRP1 atg8Δ::His3*	This study	Fig. S3
YLY10927	SEY6210 *VPS38-mNeonGreen-KanMX3 SNF7-mCherry-TRP1 atg21Δ::hphMX4*	This study	Fig. S3
YLY10928	SEY6210 *VPS38-mNeonGreen-KanMX3 SNF7-mCherry-TRP1 atg8Δ::His3 atg21Δ::hphMX4*	This study	Fig. S3
YLY10924	SEY6210 *VPS38-mNeonGreen-KanMX3 SNF7-mCherry-TRP1 vps21Δ::hphMX4*	This study	Fig. S3
YLY10926	SEY6210 *VPS38-mNeonGreen-KanMX3 SNF7-mCherry-TRP1 vps21Δ::hphMX4 atg8Δ::His3*	This study	Fig. S3
YLY10929	SEY6210 *VPS38-mNeonGreen-KanMX3 SNF7-mCherry-TRP1 atg1Δ::hphMX4*	This study	Fig. S3
YLY10576	SEY6210 *VPS34-mNeonGreen-KanMX3 SNF7-mCherry-TRP1 atg8Δ::His3*	This study	Fig. S4
YLY10596	SEY6210 *VPS34-mNeonGreen-KanMX3 SNF7-mCherry-TRP1 atg21Δ::hphMX4 atg8Δ::His3*	This study	Fig. S4
YLY10577	SEY6210 *VPS34-mNeonGreen-KanMX3 SNF7-mCherry-TRP1 vps21Δ::hphMX4 atg8Δ::His3*	This study	Fig. S4
YLY10592	SEY6210 *VPS34-mNeonGreen-KanMX3 SNF7-mCherry-TRP1 atg1Δ::hphMX4*	This study	Fig. S4
YLY3043	SEY6210 *Atg14-*2x*mNeonGreen-KanMX3 SNF7-mCherry-TRP1*	This study	Fig. S5
YLY11250	SEY6210 *Atg14-*2x*mNeonGreen-KanMX3 SNF7-mCherry-TRP1 atg8Δ::His3*	This study	Fig. S5
YLY10827	SEY6210 *Atg14-*2x*mNeonGreen-KanMX3 SNF7-mCherry-TRP1 atg21Δ::hphMX4*	This study	Fig. S5
YLY10828	SEY6210 *Atg14-*2x*mNeonGreen-KanMX3 SNF7-mCherry-TRP1 atg21Δ::hphMX4 atg8Δ::His3*	This study	Fig. S5
YLY10824	SEY6210 *Atg14-*2x*mNeonGreen-KanMX3 SNF7-mCherry-TRP1 vps21Δ::hphMX4*	This study	Fig. S5
YLY10829	SEY6210 *Atg14-*2x*mNeonGreen-KanMX3 SNF7-mCherry-TRP1 vps21Δ::hphMX4 atg8Δ::His3*	This study	Fig. S5

YLY10830	SEY6210 *Atg14*-2x*mNeonGreen-KanMX3 SNF7-mCherry-TRP1 atg1Δ::hphMX4*	This study	Fig. S5
YLY10495	SEY6210 *VPS38-mNeonGreen-KanMX3 leu2::RFP-APE1-LEU2 atg8Δ::His3*	This study	Fig. S6A, D
YLY10497	SEY6210 *VPS38-mNeonGreen-KanMX3 leu2::RFP-APE1-LEU2 atg21Δ::hphMX4*	This study	Fig. S6A, D
YLY10498	SEY6210 *VPS38-mNeonGreen-KanMX3 leu2::RFP-APE1-LEU2 atg21Δ::hphMX4 atg8Δ::His3*	This study	Fig. S6A, D
YLY10496	SEY6210 *VPS38-mNeonGreen-KanMX3 leu2::RFP-APE1-LEU2 vps21Δ::hphMX4 atg8Δ::His3*	This study	Fig. S6A, D
YLY10499	SEY6210 *VPS38-mNeonGreen-KanMX3 leu2::RFP-APE1-LEU2 atg1Δ::hphMX4*	This study	Fig. S6A, D
YLY10479	SEY6210 *VPS34-mNeonGreen-KanMX3 leu2::RFP-APE1-LEU2 atg8Δ::His3*	This study	Fig. S6B, E, S9C
YLY10481	SEY6210 *VPS34-mNeonGreen-KanMX3 leu2::RFP-APE1-LEU2 atg21Δ::hphMX4*	This study	Fig. S6B, E, S9A
YLY10482	SEY6210 *VPS34-mNeonGreen-KanMX3 leu2::RFP-APE1-LEU2 atg8Δ::His3 atg21Δ::hphMX4*	This study	Fig. S6B, E
YLY10478	SEY6210 *VPS34-mNeonGreen-KanMX3 leu2::RFP-APE1-LEU2 vps21Δ::hphMX4*	This study	Fig. S6B, E
YLY10480	SEY6210 *VPS34-mNeonGreen-KanMX3 leu2::RFP-APE1-LEU2 vps21Δ::hphMX4 atg8Δ::His3*	This study	Fig. S6B, E
YLY10483	SEY6210 *VPS34-mNeonGreen-KanMX3 leu2::RFP-APE1-LEU2 atg1Δ::hphMX4*	This study	Fig. S6B, E
YLY10820	SEY6210 *Atg14*-2x*mNeonGreen-KanMX3 leu2::RFP-APE1-LEU2 atg8Δ::His3*	This study	Fig. S6C, F
YLY10822	SEY6210 *Atg14*-2x*mNeonGreen-KanMX3 leu2::RFP-APE1-LEU2 atg21Δ::hphMX4*	This study	Fig. S6C, F
YLY10825	SEY6210 *Atg14*-2x*mNeonGreen-KanMX3 leu2::RFP-APE1-LEU2 atg21Δ::hphMX4 atg8Δ::His3*	This study	Fig. S6C, F
YLY10821	SEY6210 *Atg14*-2x*mNeonGreen-KanMX3 leu2::RFP-APE1-LEU2 atg8Δ::His3 vps21Δ::hphMX4*	This study	Fig. S6C, F
YLY10826	SEY6210 *Atg14*-2x*mNeonGreen-KanMX3 leu2::RFP-APE1-LEU2 atg1Δ::hphMX4*	This study	Fig. S6C, F

YLY8099	SEY6210 *ura3::GFP-Atg8-URA3 vps8Δ::hphMX4*	This study	Fig. S7A
YLY8031	SEY6210 *vps34Δ::hphMX4* + pRS425-Met5-DsRed-FYVE	This study	Fig. S7B
YLY3735	SEY6210 *ura3::GFP-Atg8-URA3 vps39Δ::KanMX3*	This study	Fig. S7C
YLY3459	SEY6210 *trp1::RFP-APE1-TRP1 atg21Δ::hphMX4*	This study	Fig. S7D
YLY2422	SEY6210 *ura3::GFP-Atg8-URA3*	[20]	Fig. S7E-F
YLY6405	SEY6210 *ura3::GFP-Atg8-URA3 snf7Δ::KanMX3*	[22]	Fig. S7E-F
YLY10834	SEY6210 *ura3::GFP-Atg8-URA3 SNF7-2xmTagBFP2::TRP1*	This study	Fig. S7E-F
YLY10835	SEY6210 *ura3::GFP-Atg8-URA3 snf7Δ::KanMX3 SNF7-2xmTagBFP2::TRP1*	This study	Fig. S7E-F
YLY10200	SEY6210 *Atg21-mNeonGreen-KanMX3 SNF7-mCherry-TRP1 atg8Δ::hphMX4*	This study	Fig. S8A, C
YLY10400	SEY6210 *Atg21-mNeonGreen-KanMX3 SNF7-mCherry-TRP1 atg16Δ::His3*	This study	Fig. S8A, C
YLY10402	SEY6210 *Atg21-mNeonGreen-KanMX3 SNF7-mCherry-TRP1 atg8Δ::hphMX4 atg16Δ::His3*	This study	Fig. S8A, C
YLY10404	SEY6210 *Atg21-mNeonGreen-KanMX3 SNF7-mCherry-TRP1 atg8Δ::hphMX4 vps21Δ::Lys*	This study	Fig. S8A, C
YLY10278	SEY6210 *Atg21-mNeonGreen-KanMX3 SNF7-mCherry-TRP1 atg1Δ::hphMX4*	This study	Fig. S8A, C
YLY9471	SEY6210 *Atg21-mNeonGreen-KanMX3 leu2::RFP-APE1-LEU2*	This study	Fig. S8B, D
YLY9843	SEY6210 *Atg21-mNeonGreen-KanMX3 leu2::RFP-APE1-LEU2 atg8Δ::hphMX4*	This study	Fig. S8B, D
YLY10399	SEY6210 *Atg21-mNeonGreen-KanMX3 leu2::RFP-APE1-LEU2 atg16Δ::His3*	This study	Fig. S8B, D
YLY10401	SEY6210 *Atg21-mNeonGreen-KanMX3 leu2::RFP-APE1-LEU2 atg8Δ::hphMX4 atg16Δ::His3*	This study	Fig. S8B, D
YLY10322	SEY6210 *Atg21-mNeonGreen-KanMX3 leu2::RFP-APE1-LEU2 vps21Δ::Lys*	This study	Fig. S8B, D
YLY10403	SEY6210 *Atg21-mNeonGreen-KanMX3*	This study	Fig. S8B, D

	leu2::RFP-APE1-LEU2 atg8Δ::hphMX4 vps21Δ::Lys		
YLY9501	SEY6210 *Atg21-mNeonGreen-KanMX3 leu2::RFP-APE1-LEU2 atg1Δ::hphMX4*	This study	Fig. S8B, D
YLY9725	SEY6210 *leu2::RFP-APE1-LEU2 atg16Δ::hphMX4*	This study	Fig. S9B
YLY8190	SEY6210 *ATG8::mCherry-ATG8-TRP1*	This study	Fig. S9D-E
YLY10685	SEY6210 *ATG8::mCherry-ATG8-TRP1 atg12Δ::hphMX4*	This study	Fig. S9D-E
YLY3556	SEY6210 *trp1::RFP-APE1-TRP1 atg16Δ::His3*	This study	Fig. S9F
YLY10819	SEY6210 *trp1::RFP-APE1-TRP1 atg5Δ::His3*	This study	Fig. S9G-H

B. Plasmids

Plasmid	Alias	Genotype	Source
pYL882		pFA6a-mNeonGreen-*KanMX3*	Addgene
pYL977		pFA6a-tdTomato-*TRP1*	This study
pYL1252	Snf7-mCherry	ClhN-*SNF7*-mCherry-*TRP1*	[22]
pYL482		pFA6a-VC-*KanMX36*	[70]
pYL479		pFA6a-VN-*His3MX*	[70]
pYL1108		pRS304-mCherry-Atg8	[71]
pYL267		pGBKT7	Clontech
pYL450		pGBKT7-Vps34	This study
pYL677		pGBKT7-Vps39	This study
pYL175		pACT2	This study
pYL291		pACT2-Vps8	This study
pYL250		pGADT7	Clontech
pYL529		pGADT7-Atg21	This study
pYL1460		pFA6a-2mNeonGreen-KanMX3	This study
pYL1507		ClhN-*SEC63*-2xmTagBFP2-*TRP1*	[72]

pYL1509		ClhN-*SNF7*-2xmTagBFP2-*TRP1*	This study
pYL237		pRS304-RFP-Ape1	This study
pYL238		pRS305-RFP-Ape1	[66]
pYL1510		ClhN-*SNF7*-2xmTagBFP2-*LEU2*	This study
pYL502	pVC	pUG34-Venus-C (*HIS3*)	[67]
pYL1186		pVC-Atg21	This study
pYL501	pVN	pUG36-Venus-N (*URA3*)	[67]
pYL1209		pVN-Atg16	This study
pYL1208		pVN-Atg8	This study
pYL1407		pRS415-*CUP1p*-yEGFP	This study
pYL1431		pRS415-*CUP1p*-yEGFP-Atg12	This study
pYL1005		pRS415-*CUP1p*-yEGFP-Atg16	This study
pYL1518		pRS415-*CUP1p*-Atg5-yEGFP	This study
pYL173		pRS425-*MET3p*-DsRed-FYVE	[73]
pYL292		pFBT9-Vps21 WT	[74]
pYL935		pFBT9-Vps21S21N(GDP)	[74]
pYL987		pFBT9-Vps21Q66L(GTP)	[74]
pYL720		pGBKT7-Vps8	This study
pYL254		pRS415-*CUP1p*-mCherry-Atg8	[75]
pYL1203		pVC-Atg16	This study
