# Supplementary material for: Vps21 Directs the PI3K-PI(3)P-Atg21-Atg16 Module to Phagophores via Vps8 for Autophagy
Source: Int J Mol Sci. 2022 Aug 23;23(17):9550. doi: 10.3390/ijms23179550 (PMC9455592; doi:10.3390/ijms23179550)
Supplement: Supplementary file 1 [file ijms-23-09550-s001.zip › ijms-1879267-supplementary/R1-4-082022-IJMS-Supp Figures in PDF.pdf]

Figure S1

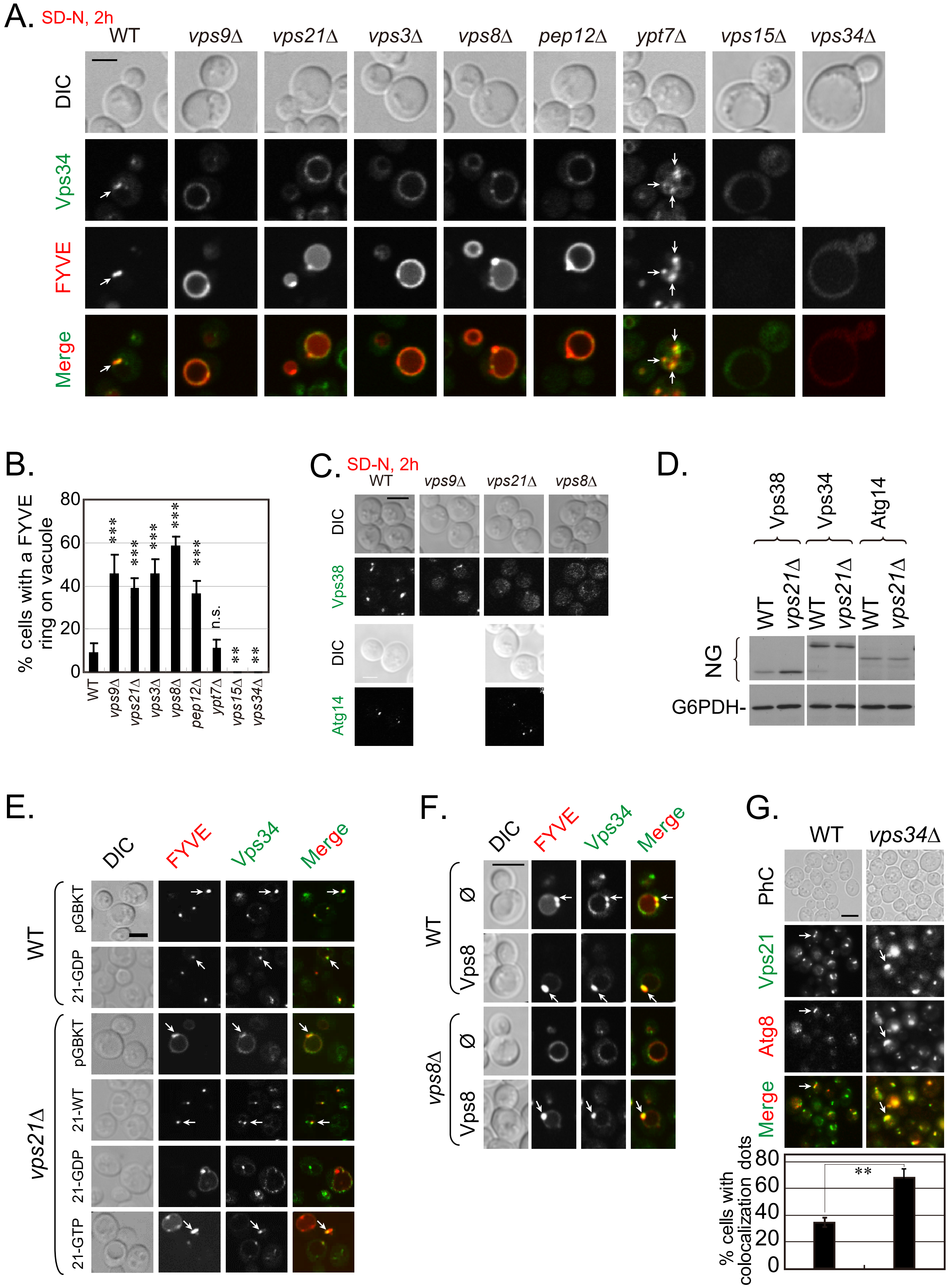
A.
SD-N, 2h
WT
vps9Δ
vps21Δ
vps3Δ
vps8Δ
pep12Δ
ypt7Δ
vps15Δ
vps34Δ
DIC
Vps34
FYVE
Merge
B.
% cells with a FYVE ring on vacuole
80
60
40
20
0
***
n.s.
**
WT
vps9Δ
vps21Δ
vps3Δ
vps8Δ
pep12Δ
ypt7Δ
vps15Δ
vps34Δ
C.
SD-N, 2h
WT
vps9Δ
vps21Δ
vps8Δ
DIC
Vps38
Atg14
D.
Vps38
Vps34
Atg14
WT
vps21Δ
NG
G6PDH-
E.
DIC
FYVE
Vps34
Merge
WT
vps21Δ
pGBKT
21-GDP
21-WT
21-GTP
F.
DIC
FYVE
Vps34
Merge
WT
vps8Δ
Ø
Vps8
G.
WT
vps34Δ
PhC
Vps21
Atg8
Merge
% cells with colocalization dots
80
60
40
20
0
**

Figure S2

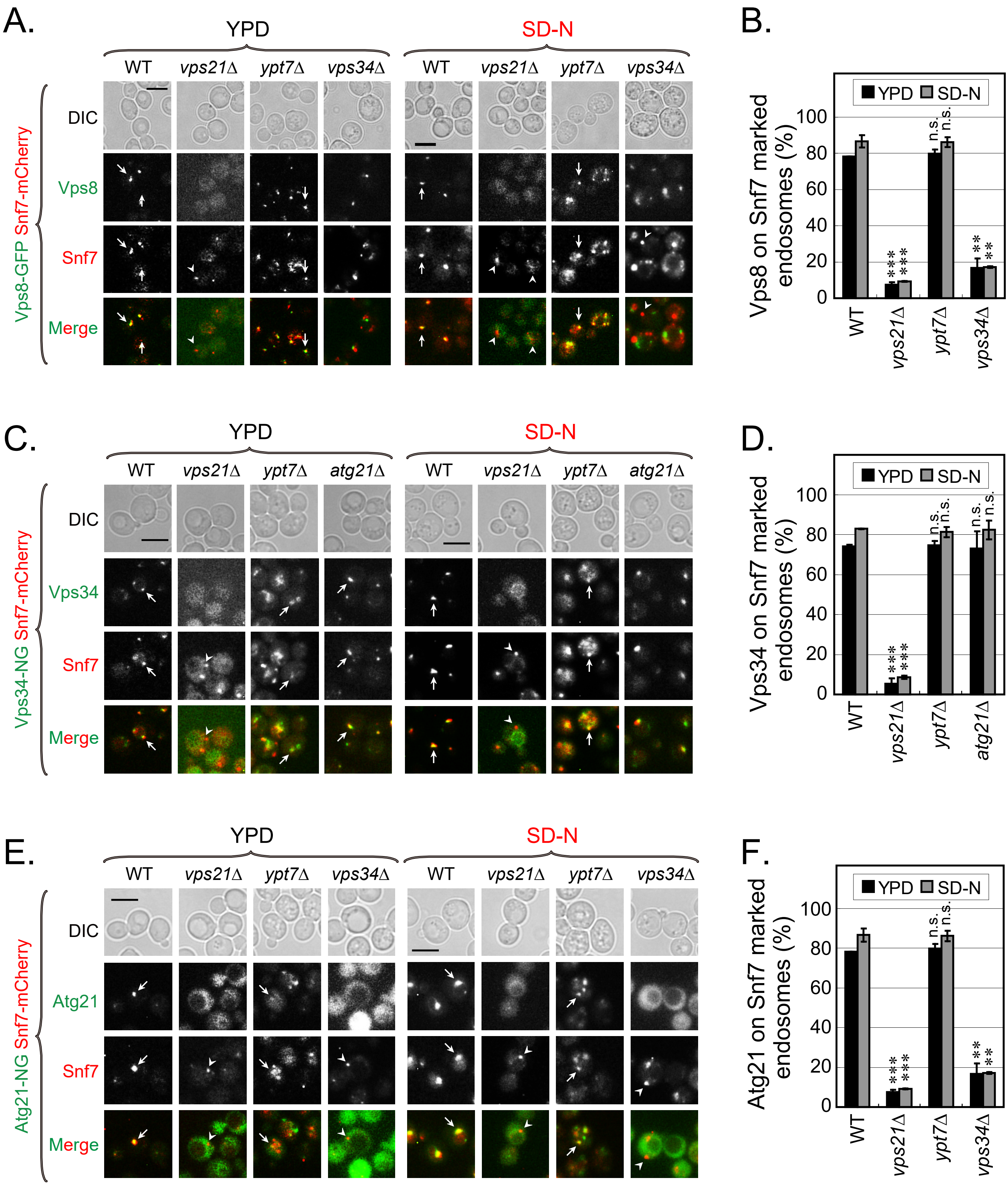

A.
YPD
SD-N
WT
vps21Δ
ypt7Δ
vps34Δ
Vps8-GFP Snf7-mCherry
DIC
Vps8
Snf7
Merge
B.
YPD
SD-N
Vps8 on Snf7 marked endosomes (%)
0
20
40
60
80
100
n.s.
***
**
WT
vps21Δ
ypt7Δ
vps34Δ
C.
YPD
SD-N
WT
vps21Δ
ypt7Δ
atg21Δ
Vps34-NG Snf7-mCherry
DIC
Vps34
Snf7
Merge
D.
YPD
SD-N
Vps34 on Snf7 marked endosomes (%)
0
20
40
60
80
100
n.s.
***
WT
vps21Δ
ypt7Δ
atg21Δ
E.
YPD
SD-N
WT
vps21Δ
ypt7Δ
vps34Δ
Atg21-NG Snf7-mCherry
DIC
Atg21
Snf7
Merge
F.
YPD
SD-N
Atg21 on Snf7 marked endosomes (%)
0
20
40
60
80
100
n.s.
***
**
WT
vps21Δ
ypt7Δ
vps34Δ

Figure S3

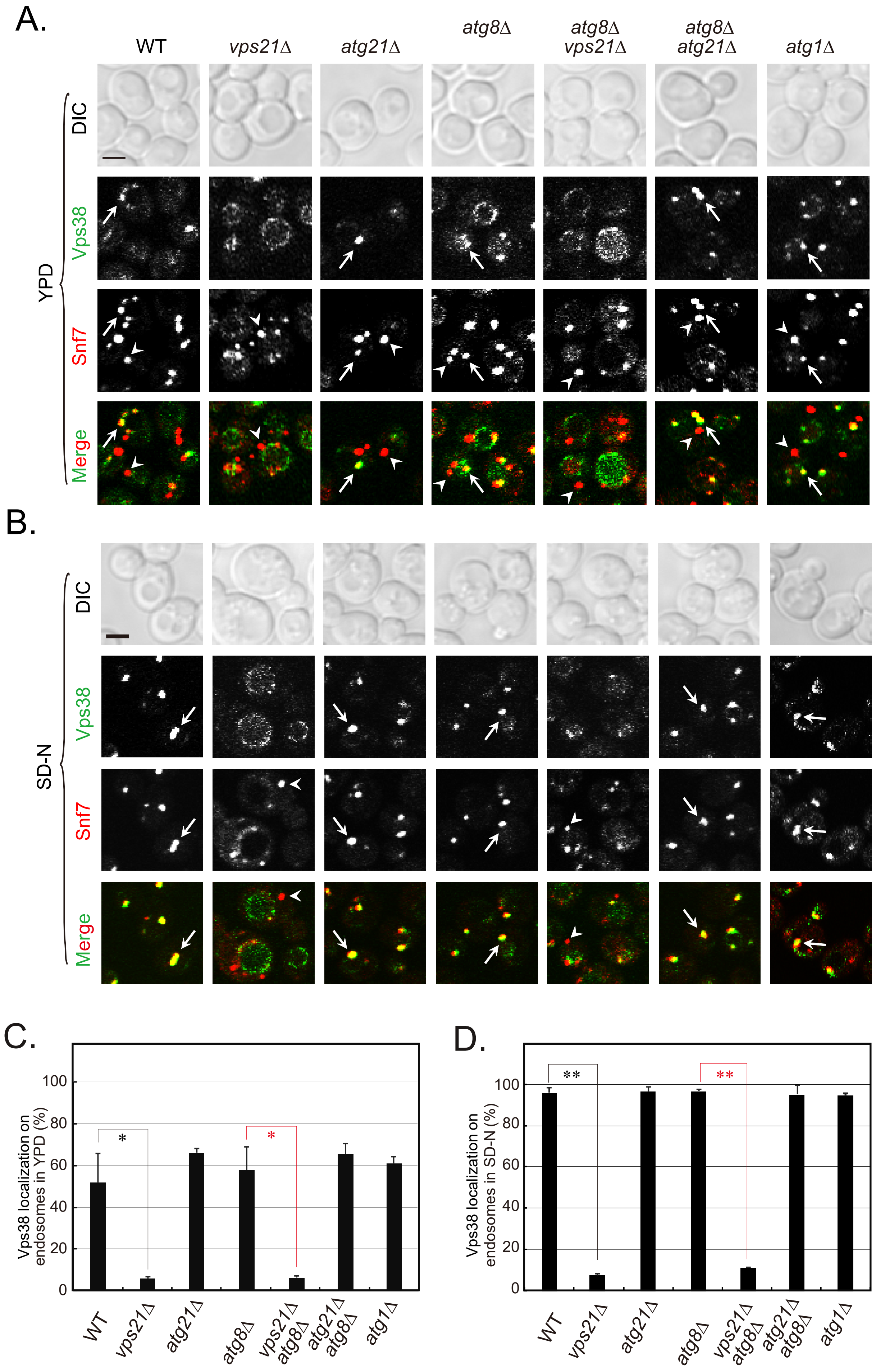

A.
WT
vps21Δ
atg21Δ
atg8Δ
atg8Δ vps21Δ
atg8Δ atg21Δ
atg1Δ
YPD
DIC
Vps38
Snf7
Merge
B.
SD-N
DIC
Vps38
Snf7
Merge
C.
Vps38 localization on endosomes in YPD (%)
0
20
40
60
80
100
*
*
WT
vps21Δ
atg21Δ
atg8Δ
vps21Δ atg8Δ
atg21Δ atg8Δ
atg1Δ
D.
Vps38 localization on endosomes in SD-N (%)
0
20
40
60
80
100
**
**
WT
vps21Δ
atg21Δ
atg8Δ
vps21Δ atg8Δ
atg21Δ atg8Δ
atg1Δ

Figure S4

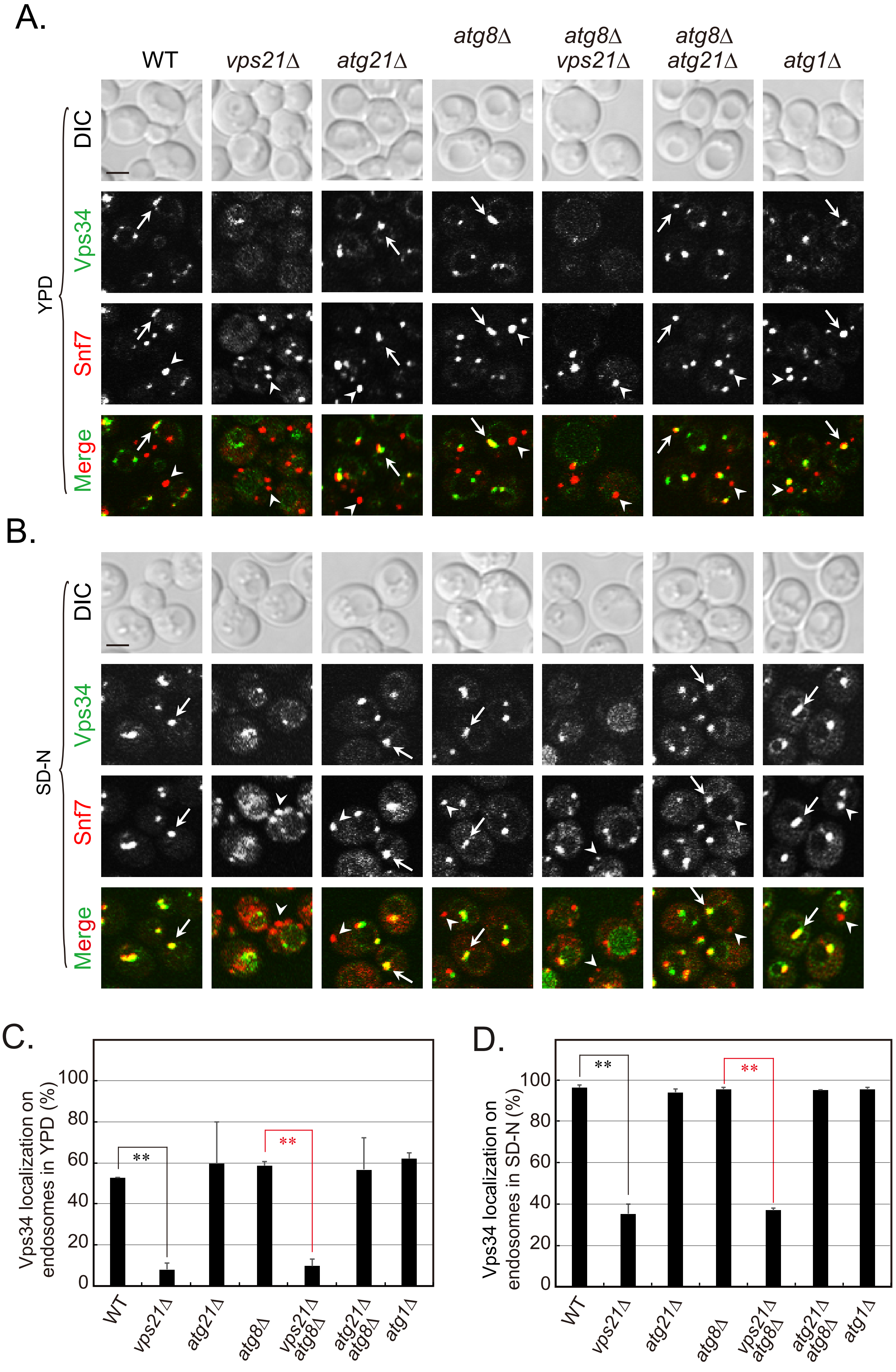

A.
WT
vps21Δ
atg21Δ
atg8Δ
atg8Δ vps21Δ
atg8Δ atg21Δ
atg1Δ
DIC
Vps34
Snf7
Merge
YPD
B.
SD-N
C.
Vps34 localization on endosomes in YPD (%)
0
20
40
60
80
100
**
**
WT
vps21Δ
atg21Δ
atg8Δ
vps21Δ atg8Δ
atg21Δ atg8Δ
atg1Δ
D.
Vps34 localization on endosomes in SD-N (%)

Figure S5

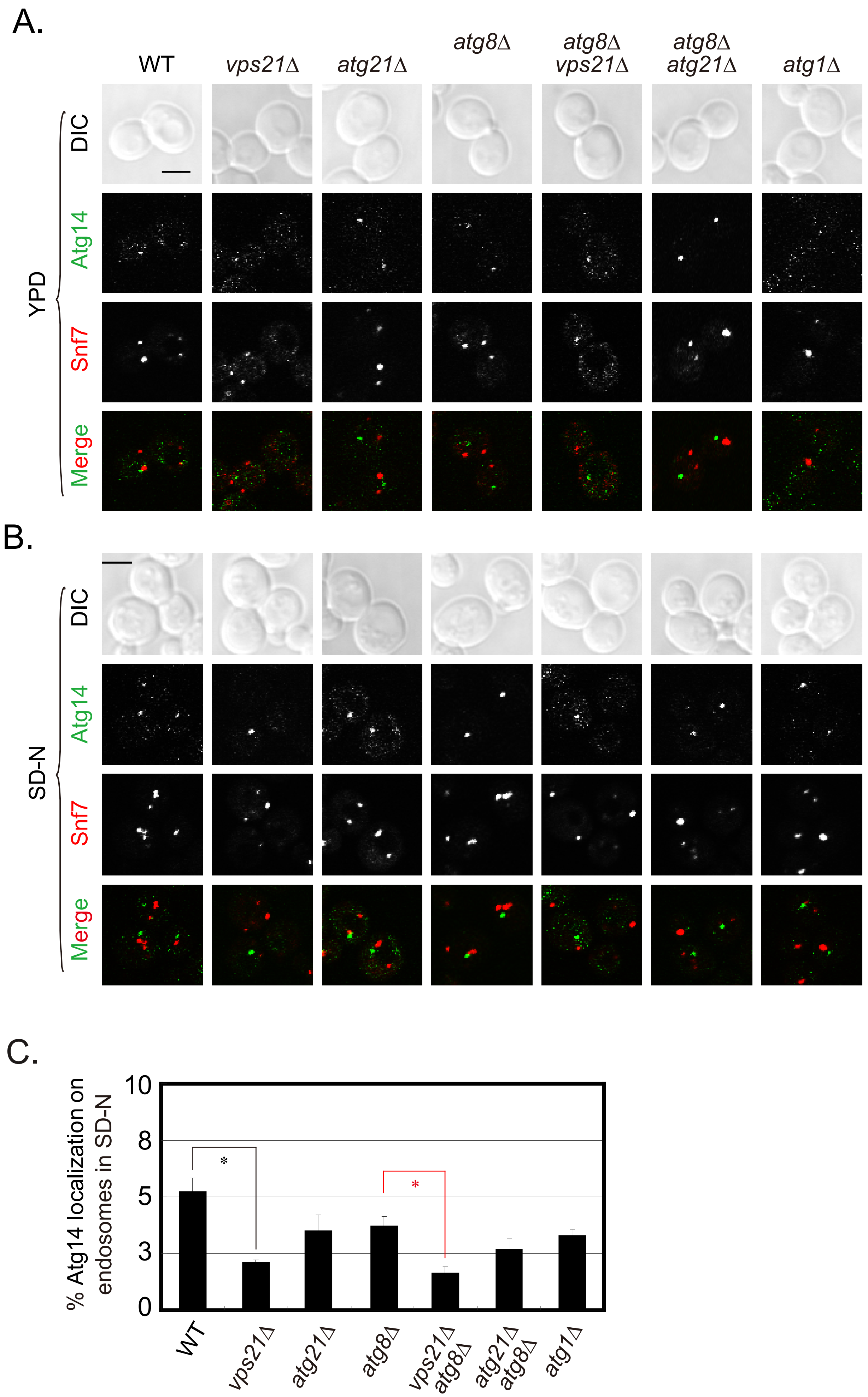
A.
WT
vps21Δ
atg21Δ
atg8Δ
atg8Δ vps21Δ
atg8Δ atg21Δ
atg1Δ
YPD
DIC
Atg14
Snf7
Merge
B.
SD-N
DIC
Atg14
Snf7
Merge
C.
% Atg14 localization on endosomes in SD-N
10
8
5
3
0
*
*
WT
vps21Δ
atg21Δ
atg8Δ
vps21Δ atg8Δ
atg21Δ atg8Δ
atg1Δ

Figure S6

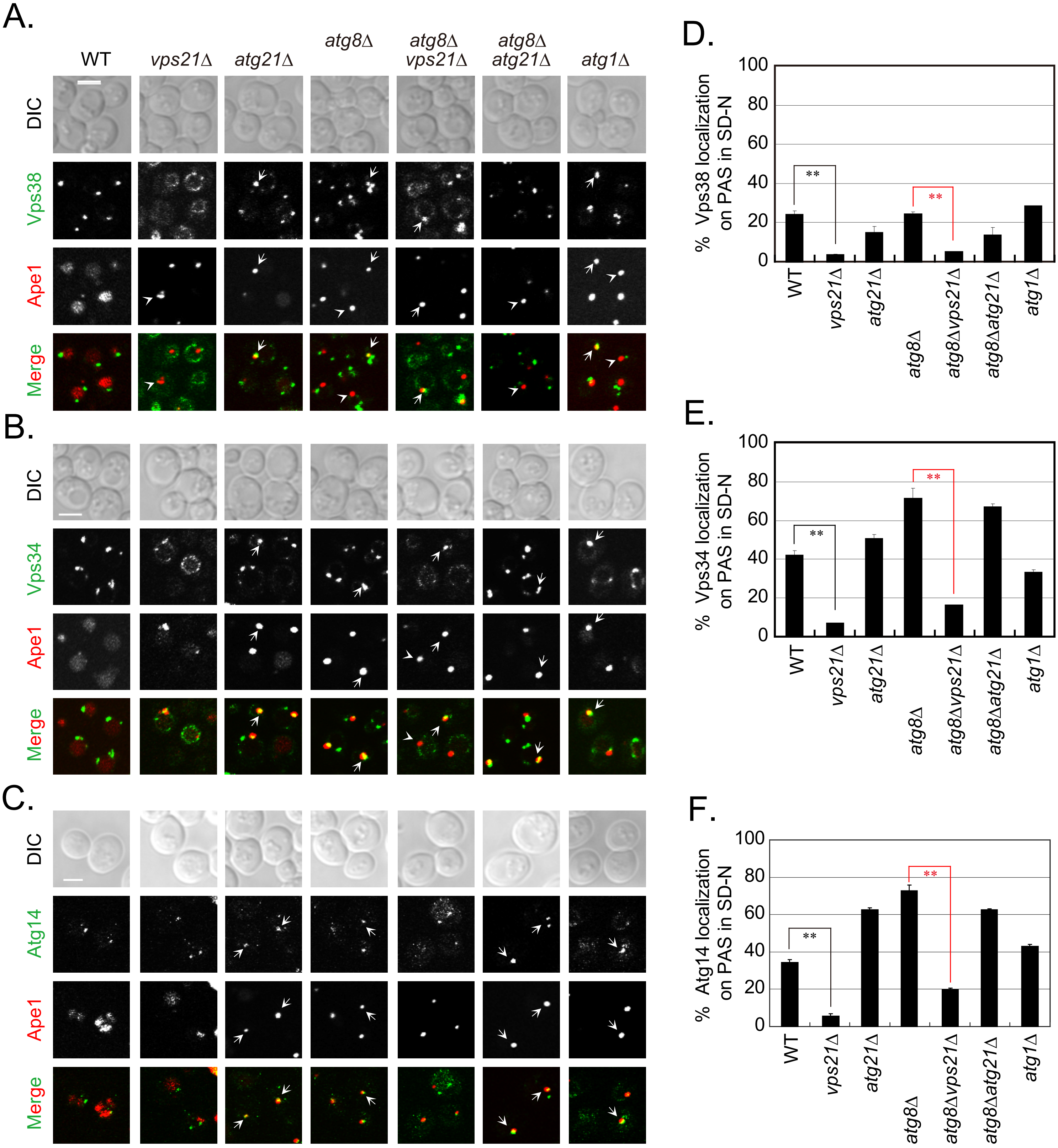

A.
WT
vps21Δ
atg21Δ
atg8Δ
atg8Δ vps21Δ
atg8Δ atg21Δ
atg1Δ
DIC
Vps38
Ape1
Merge
B.
DIC
Vps34
Ape1
Merge
C.
DIC
Atg14
Ape1
Merge
D.
% Vps38 localization on PAS in SD-N
100
80
60
40
20
0
**
**
WT
vps21Δ
atg21Δ
atg8Δ
atg8Δvps21Δ
atg8Δatg21Δ
atg1Δ
E.
% Vps34 localization on PAS in SD-N
100
80
60
40
20
0
**
**
WT
vps21Δ
atg21Δ
atg8Δ
atg8Δvps21Δ
atg8Δatg21Δ
atg1Δ
F.
% Atg14 localization on PAS in SD-N
100
80
60
40
20
0
**
**
WT
vps21Δ
atg21Δ
atg8Δ
atg8Δvps21Δ
atg8Δatg21Δ
atg1Δ

Figure S7

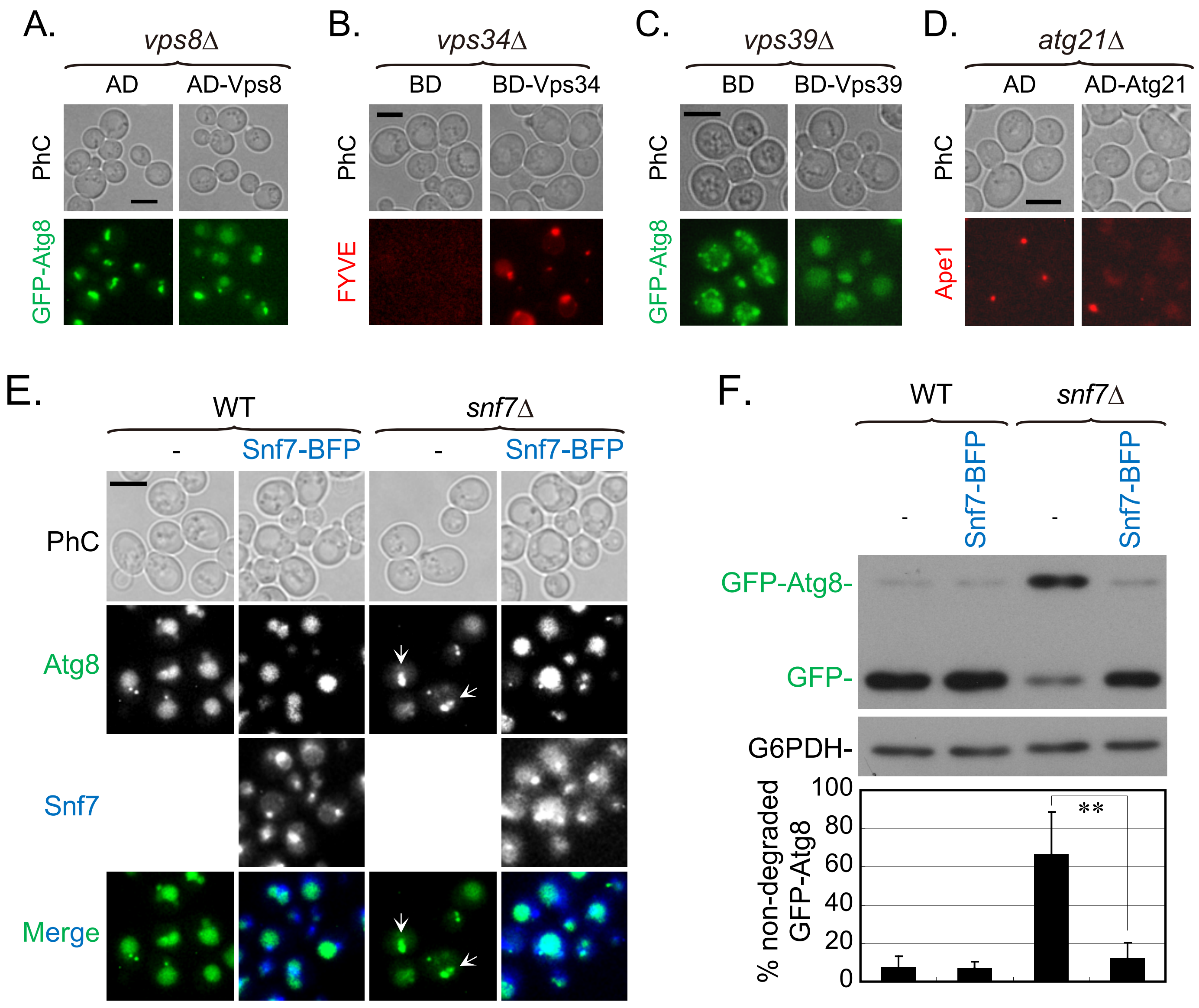

A.
vps8Δ
AD
AD-Vps8
PhC
GFP-Atg8
B.
vps34Δ
BD
BD-Vps34
PhC
FYVE
C.
vps39Δ
BD
BD-Vps39
PhC
GFP-Atg8
D.
atg21Δ
AD
AD-Atg21
PhC
Ape1
E.
WT
snf7Δ
-
Snf7-BFP
-
Snf7-BFP
PhC
Atg8
Snf7
Merge
F.
WT
snf7Δ
-
Snf7-BFP
-
Snf7-BFP
GFP-Atg8-
GFP-
G6PDH-
% non-degraded GFP-Atg8
100
80
60
40
20
0
**

Figure S8

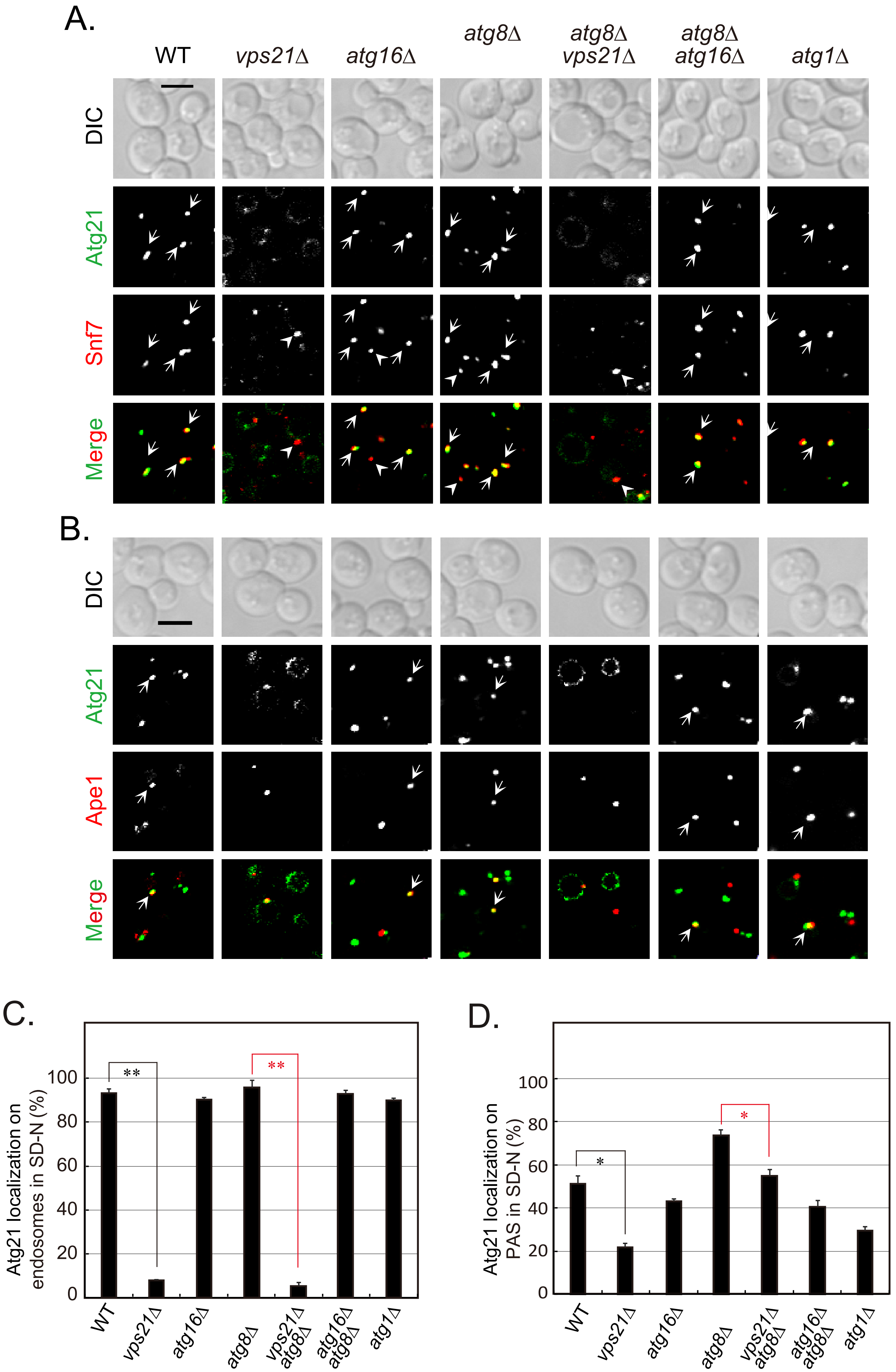
A.
WT
vps21Δ
atg16Δ
atg8Δ
atg8Δ vps21Δ
atg8Δ atg16Δ
atg1Δ
DIC
Atg21
Snf7
Merge
B.
DIC
Atg21
Ape1
Merge
C.
Atg21 localization on endosomes in SD-N (%)
**
**
0
20
40
60
80
100
WT
vps21Δ
atg16Δ
atg8Δ
vps21Δ atg8Δ
atg16Δ atg8Δ
atg1Δ
D.
Atg21 localization on PAS in SD-N (%)
*
*
0
20
40
60
80
100
WT
vps21Δ
atg16Δ
atg8Δ
vps21Δ atg8Δ
atg16Δ atg8Δ
atg1Δ

Figure S9

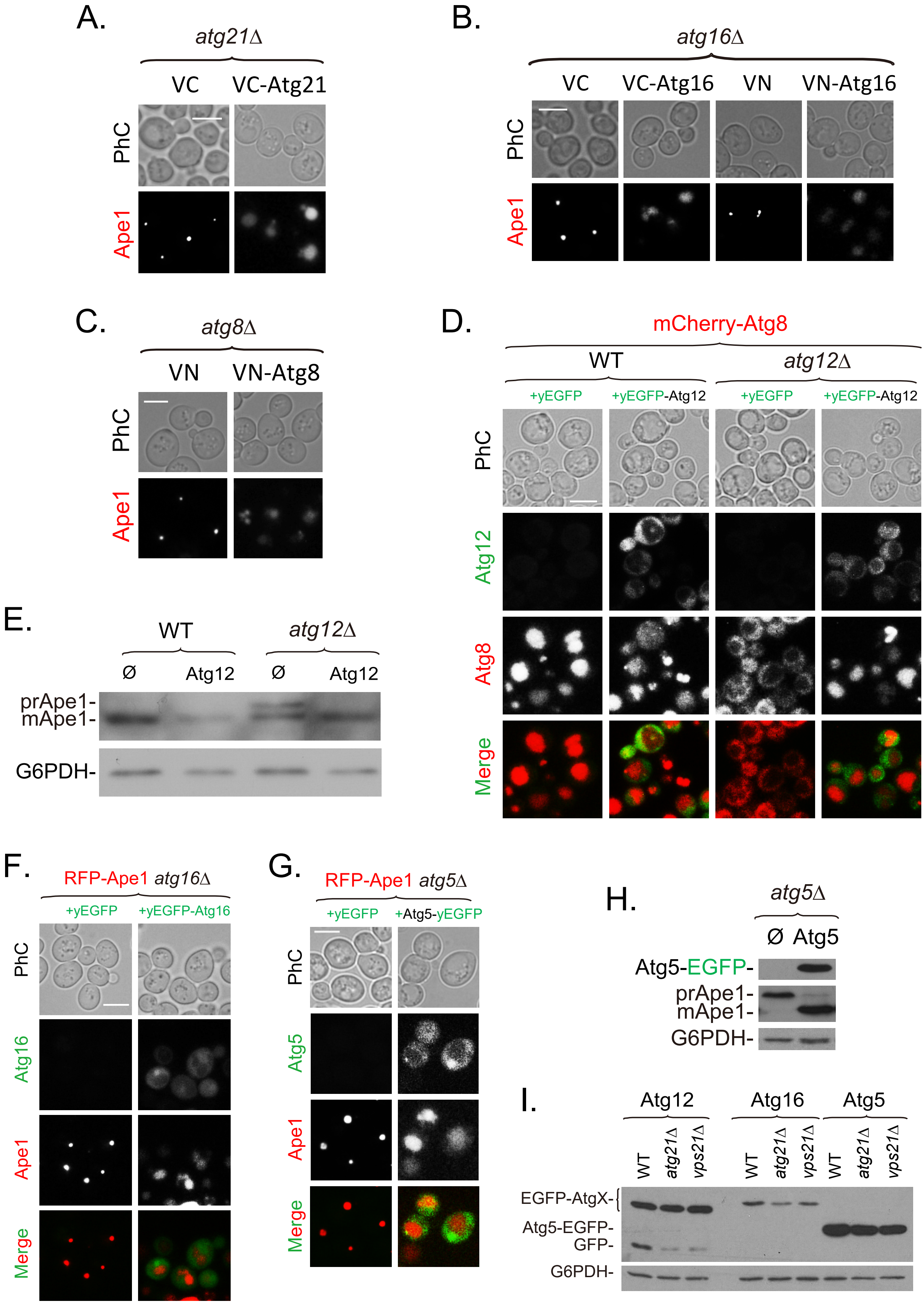
A.
atg21Δ
VC
VC-Atg21
PhC
Ape1
B.
atg16Δ
VC
VC-Atg16
VN
VN-Atg16
PhC
Ape1
C.
atg8Δ
VN
VN-Atg8
PhC
Ape1
D.
mCherry-Atg8
WT
atg12Δ
+yEGFP
+yEGFP-Atg12
+yEGFP
+yEGFP-Atg12
PhC
Atg12
Atg8
Merge
E.
WT
atg12Δ
Ø
Atg12
Ø
Atg12
prApe1-
mApe1-
G6PDH-
F.
RFP-Ape1 atg16Δ
+yEGFP
+yEGFP-Atg16
PhC
Atg16
Ape1
Merge
G.
RFP-Ape1 atg5Δ
+yEGFP
+Atg5-yEGFP
PhC
Atg5
Ape1
Merge
H.
atg5Δ
Ø Atg5
Atg5-EGFP-
prApe1-
mApe1-
G6PDH-
I.
Atg12
Atg16
Atg5
WT
atg21Δ
vps21Δ
WT
atg21Δ
vps21Δ
WT
atg21Δ
vps21Δ
EGFP-AtgX-
Atg5-EGFP-
GFP-
G6PDH-
